# Loss of DDB1 Leads to Transcriptional p53 Pathway Activation in Proliferating Cells, Cell Cycle Deregulation, and Apoptosis in Zebrafish Embryos

**DOI:** 10.1371/journal.pone.0134299

**Published:** 2015-07-30

**Authors:** Zhilian Hu, Jochen Holzschuh, Wolfgang Driever

**Affiliations:** 1 Developmental Biology, Institute Biology I, Faculty of Biology, Albert-Ludwigs-University Freiburg, Hauptstrasse 1, 79104, Freiburg, Germany; 2 Department of Pediatrics and Communicable Diseases, University of Michigan, Ann Arbor, MI, 48109–5646, United States of America; 3 BIOSS—Centre for Biological Signalling Studies, Albert-Ludwigs-University Freiburg, Schänzlestrasse 18, 79104, Freiburg, Germany; Wayne State University School of Medicine, UNITED STATES

## Abstract

DNA damage-binding protein 1 (DDB1) is a large subunit of the heterodimeric DDB complex that recognizes DNA lesions and initiates the nucleotide excision repair process. DDB1 is also a component of the CUL4 E3 ligase complex involved in a broad spectrum of cellular processes by targeted ubiquitination of key regulators. Functions of DDB1 in development have been addressed in several model organisms, however, are not fully understood so far. Here we report an ENU induced mutant *ddb1* allele (*ddb1^m863^*) identified in zebrafish (*Danio rerio*), and analyze its effects on development. Zebrafish *ddb1* is expressed broadly, both maternally and zygotically, with enhanced expression in proliferation zones. The (*ddb1^m863^* mutant allele affects the splice acceptor site of exon 20, causing a splicing defect that results in truncation of the 1140 amino acid protein after residue 800, lacking part of the β-propeller domain BPC and the C-terminal helical domain CTD. *ddb1^m863^* zygotic mutant embryos have a pleiotropic phenotype, including smaller and abnormally shaped brain, head skeleton, eyes, jaw, and branchial arches, as well as reduced dopaminergic neuron groups. However, early forming tissues develop normally in zygotic *ddb1^m863^* mutant embryos, which may be due to maternal rescue. In *ddb1^m863^* mutant embryos, *pcna*-expressing proliferating cell populations were reduced, concurrent with increased apoptosis. We also observed a concomitant strong up-regulation of transcripts of the tumor suppressor *p53 (tp53)* and the cell cycle inhibitor *cdkn1a (p21a/b^CIP1/WAF1^*) in proliferating tissues. In addition, transcription of cyclin genes *ccna2* and *ccnd1* was deregulated in *ddb1^m863^* mutants. Reduction of *p53* activity by anti-sense morpholinos alleviated the apoptotic phenotype in *ddb1^m863^* mutants. These results imply that Ddb1 may be involved in maintaining proper cell cycle progression and viability of dividing cells during development through transcriptional mechanisms regulating genes involved in cell cycle control and cell survival.

## Introduction

The genetic stability of a cell is constantly challenged by environmental and endogenous factors. 50,000–100,000 different damage events have been estimated to occur each day to the DNA in a single human cell [1]. Within the cell cycle, DNA damage has to be uncovered and repaired before or during genome replication to ensure integrity of the genome. Proper cell cycle progression and DNA repair are meticulously controlled by multiple factors including the DNA damage-binding protein (DDB) complex. One of its large subunits, DDB1, has initially been identified as a critical component of the nucleotide excision repair process (NER) for recognizing and removing DNA lesions induced by various mechanisms including ultraviolet (UV) light, chemical carcinogens, and oxidative stress [2–5]. DDB1 functions in DNA-damage repair via two sub-pathways, global genomic repair (GGR) through a heterodimeric complex of DDB1-DDB2, and transcription-coupled repair (TCR) through the interaction of DDB1 and Cockayne syndrome factor (CSA). The failure of NER may contribute to many diseases, including Down syndrome, Parkinson disease, and Huntington's disease [6]. Other functions of DDB1 beyond its accessory role in DNA repair have been associated with the CUL4 E3 ligase complex.

The CUL4 E3 ligase complex consists of an evolutionarily conserved Cullin4 as a scaffold, at its carboxy-terminus a RING-finger protein (ROC1) to assemble a catalytic core with E2 Ubiquitin-conjugating enzyme, and at its amino-terminus a Cullin-specific adaptor and substrate receptor [7–9]. DDB1, a multi-domain protein with three β-propeller folds (BPA, BPB, and BPC) and a C-terminal helical domain tail [8, 10], is such an adaptor acting in the CUL4 E3 ligase complex. The DDB1 BPB propeller domain binds to the N-terminus of CUL4, as well as to other WD40 proteins containing a DWD box (*D*DB1-binding and *WD*40 repeat box) [9, 11, 12]. DDB1 functions in the CUL4 E3 ligase complex to recruit substrate receptors (DCAFs—*D*DB1 and *C*ul4-*A*ssociated *F*actors) to CUL4. The resulting (DDB1-DCAF)-CUL4-(ROC1-E2) complexes will ubiquitinate and degrade the targeted protein substrates [13–15]. Substrates identified so far include CDT1 (Dup in Drosophila), p21^CIP1/WAF1^ (human and *C*. *elegans*), polymerase eta (*C*. *elegans*), PR-Set7/Set8, E2F1 (Drosophila), spd1 and spd2 (*S*. *pombe*), and p27^KIP1^ (mammalian) [16–23]. These substrates play a role in a wide range of cellular processes. Thus the involvement of DDB1 in CUL4 ligase can regulate, by targeting various substrates, multiple cellular processes such as transcription, cell cycle regulation, proliferation, histone lysine methylation, and the adjustment of cellular levels of the tumor suppressor p53 [24, 25]. Some activities of DDB1 have also been linked to developmental processes. Inactivation of DDB1 in fission yeast results in cell cycle and growth defects, increased rate of spontaneous mutations, and disruption of cell differentiation [14, 26, 27]. In mice, deficiency of DDB1 leads to apoptosis, aberrant accumulation of cell cycle regulators, and increased genomic instability [25, 28]. Similar defects are also observed in human U2OS cells, chicken DT40 B cells, in *Drosophila*, and *Arabidopsis thaliana* [29–35]. However, the embryonic lethality caused by complete DDB1 deficiency in model organism such as mice has limited research into potential functions during development. In contrast, due to maternal rescue, deficiency of zygotically expressed Ddb1 in zebrafish (*Danio rerio*) is only lethal at larval stages, thus facilitating studies of DDB1 function in zebrafish development (this paper).

Here, we report the analysis of Ddb1 functions during the zebrafish development. We isolated in an ENU mutagenesis screen the mutant *ddb1* ^*m863*^allele and characterized its effects on development. Disruption of *ddb1* in zebrafish resulted in a pleiotropic phenotype, including reduced size of organs that grow and differentiate during early larval stages, including brain and pharyngeal skeleton. Ddb1 deficiency appears to cause p53-dependent apoptosis of *pcna*-positive proliferating cells, and deregulation of cell cycle regulators including cyclins and CIP/KIP family members. These results suggest that impairment of Ddb1 contributes to the phenotypic defects of *ddb1* ^*m863*^ mutants by deregulation of programmed cell death and cell cycle.

## Results

### Morphological and neuronal phenotype of *m863* mutant embryos

**Fig 1:**
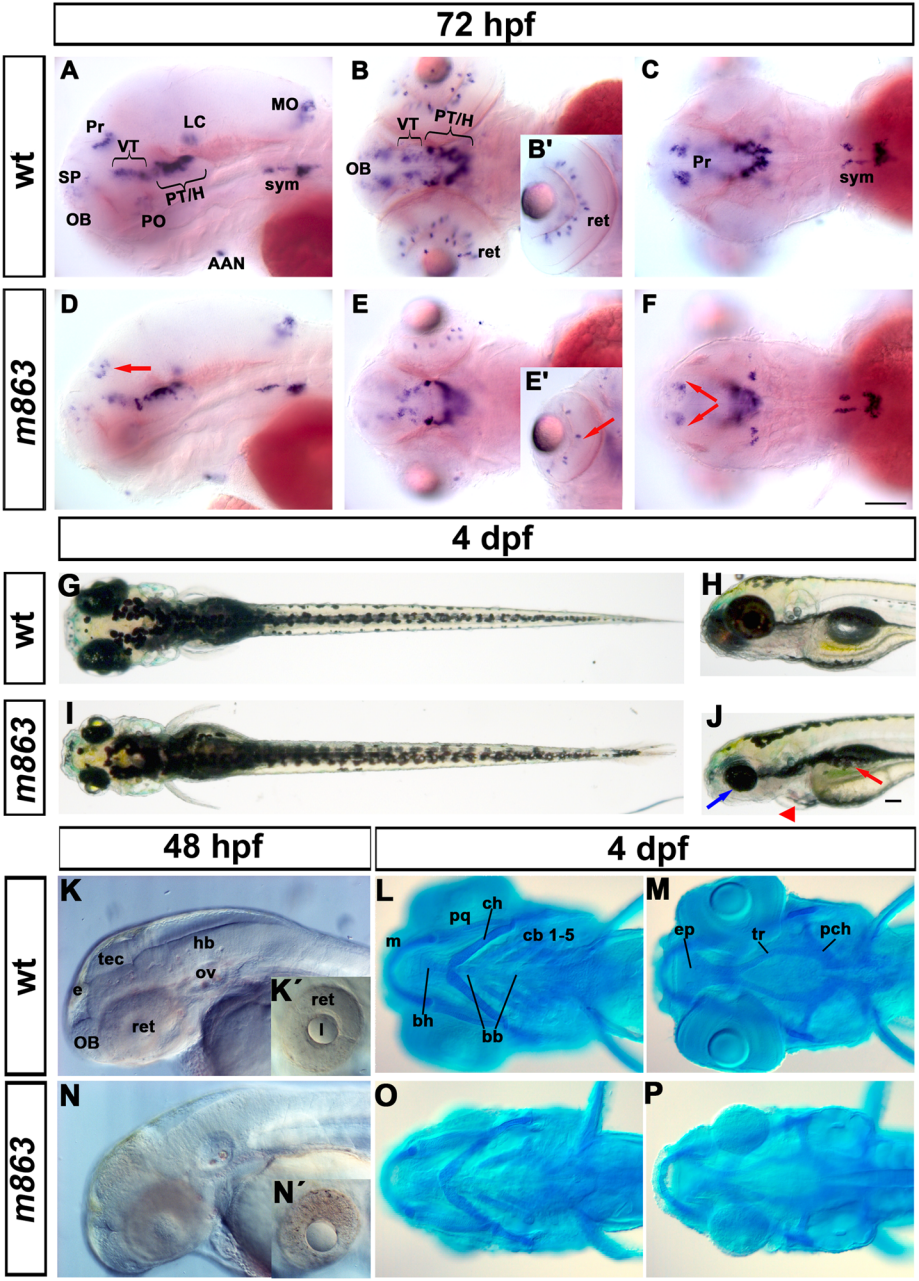
Phenotype of *m863* mutant embryos. (A-F) Reduction of *th*-expressing dopaminergic neurons in the pretectum and retina of *m863* mutants at 72hpf. (A-C) Lateral (A) and dorsal (B-C) views of *th* expression pattern in wild type siblings. (D-F) Lateral (D) and dorsal (E-F) views of *th* expression pattern in homozygous mutant *m863* larvae. Arrows indicate affected DA groups in the pretectum (D, F) and retina (E'). (G-J) Morphological phenotype of live *m863* mutants at 4 dpf. Dorsal (G) and lateral (H) views of wild type larvae. Dorsal (I) and lateral (J) views of homozygous *m863* mutants. Compared to wild type siblings, *m863* mutants displayed smaller eyes (blue arrow), flattened and smaller head, edema (red triangle), and defective swim bladder (red arrow), but normal body length. (K, N) Lateral views of live wild type (K) and homozygous *m863* mutants (N) at 48 hpf. Granular tissue appearance in the midbrain region and retina of mutants indicated elevated cell death. (L,M,O,P) Ventral views of Alcian blue staining of head cartilage in wild type (L, M) and homozygous *m863* mutants (O, P) at 4 dpf. The cartilaginous head skeleton of mutants was smaller and underdeveloped compared to wild type siblings. Abbreviations used: Catecholaminergic groups: AAN, arch-associated neurons (noradrenergic); H, hypothalamus; LC, locus coeruleus; MO, medulla oblongata (noradrenergic); OB, olfactory bulb; PO, preoptic region; Pr, pretectum; PT, posterior tuberculum; SP, subpallium; sym, sympathetic neurons (catecholaminergic); VT, ventral thalamus. Cartilage structures: bb, basibranchial; bh, basihyal; cb, ceratobranchials; ch, ceratohyal; ep, ethmoid plate; m, Meckel’s cartilage; pch, parachordal; pq, palatoquadrate; tr, trabecula. Anterior towards the left. Scale bar: 100 μm

The *m863* allele was isolated during a mutagenesis screen aimed at identifying factors involved in dopaminergic (DA) neuron differentiation in zebrafish embryos and early larvae, using *tyrosine hydroxylase* (*th*) expression as a marker. *m863* mutant embryos at 3 days post fertilization (dpf) had fewer DA neurons in the pretectum and retina, while early differentiating DA neurons of the posterior tuberculum appeared largely normal (Fig 1A–1F). Furthermore, *m863* mutant embryos from 3 dpf on displayed morphological defects, including reduced size of the brain and eyes (Fig 1G–1J). The cartilaginous head skeleton with the jaw and other branchial arch derivatives were smaller and did not develop properly in *m863* mutants (Fig 1L, 1M, 1O and 1P). In contrast, early forming tissues, including somites and notochord, developed largely normal in mutant embryos, and the body length of mutant and wild-type larvae was similar (Fig 1G–1J). Cell death, as judged from granular and turbid appearance of tissue, was already apparent in the dorsal midbrain and eyes of life *m863* mutants at 36 hpf (unpublished data). At 48 hpf, cell death appeared to be further increased in the retina and fore- and midbrain of *m863* mutant embryos, when compared to their wild-type siblings (Fig 1K and 1N). *m863* mutant larvae did not grow much beyond the phenotype observed at 4 dpf (Fig 1G–1J) and die between 5 and 10 dpf (unpublished data). The pleiotropic *m863* mutant phenotype with smaller size of tissues forming from rapidly proliferating cells during early larval development and enhanced cell death suggest that the affected gene may be involved in control of cell survival and proliferation.

### Identification of *m863* as mutant allele of the *ddb1* gene

**Fig 2:**
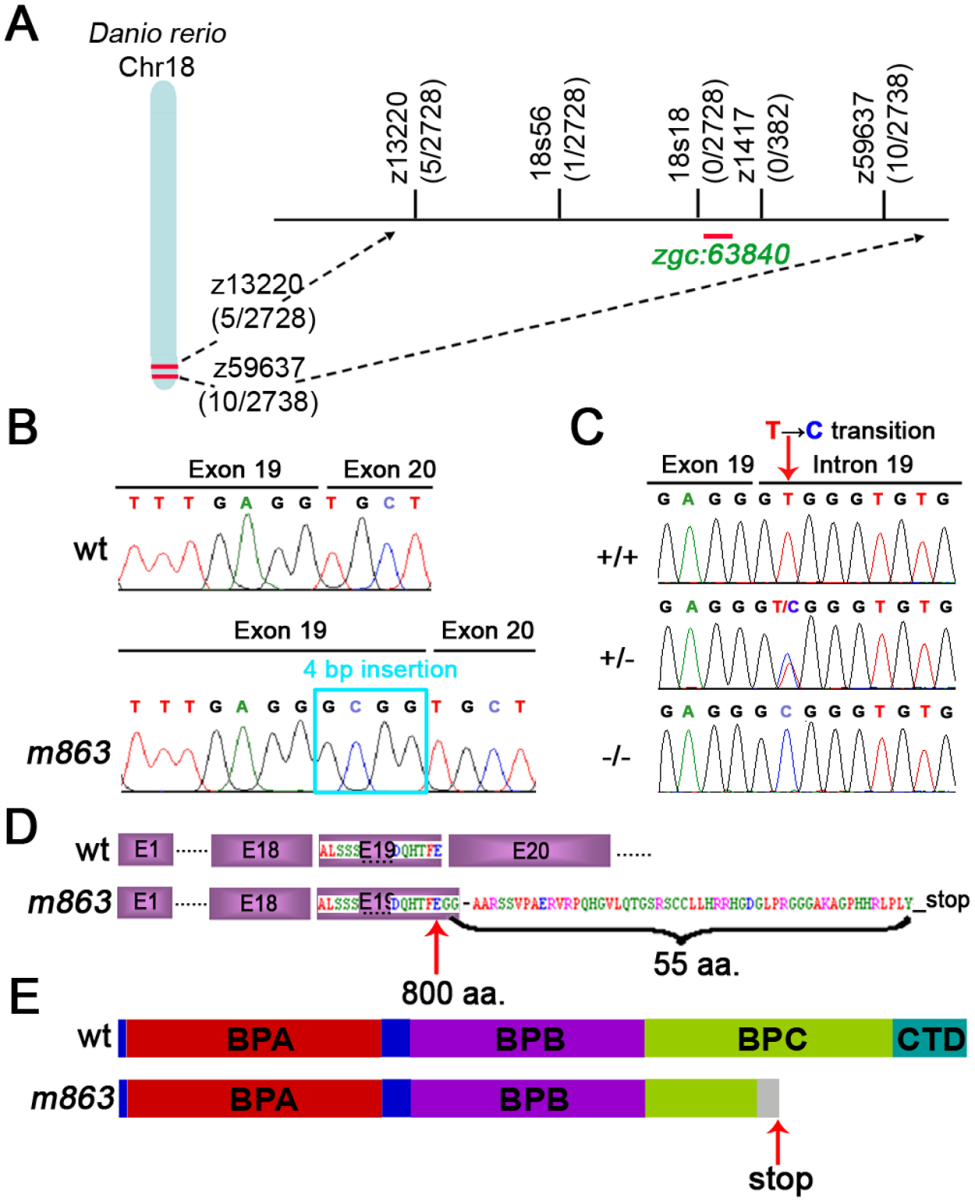
Genetic mapping and identification of the *m863* gene locus. (A) Schematic diagram of zebrafish linkage group (LG) 18 and the *m863* genomic region (Ensembl Zv8). Numbers in brackets represent recombination event per number of meiosis analyzed. The *m863* mutation was mapped to the genomic interval defined by the microsatellite markers z13220 and z59637. The SSLP marker18s56 generated in this study showed one recombinant, while no recombinants were recovered for the SSLP markers 18s18a and z1417. The gene *zgc*:*63840* mapped to the critical interval of the *m863* mutation. (B) Chromatogram of partial cDNA sequences from wild type and homozygous mutant *m863* embryos. The insertion of 4 bp occurred in *m863* embryos at position 2402–2405 (exon 19) of the *ddb1* ORF. (C) Chromatogram of genomic sequences of 3'-end of exon 19 and 5'-splice site from wild type, heterozygous embryos, and homozygous *m863* mutants. The mononucleotide substitution (T→C) in homozygous *m863* mutants affected the second core base of the splice donor consensus sequence, which is highly conserved in eukaryotic splice donor sites. (D) The frame shift by partial intron insertion introduced a stop codon after a stretch of novel 55 variant amino acids in the C-terminal part of the mutant protein. (E) Schematic diagram of the domain structure of Ddb1 protein based on human DDB1 structure prediction [10]. The partial BPC propeller is truncated and C-terminal helical domain absent in the truncated *m863* Ddb1 protein. Arrows represent the point mutation (C), the last wild type amino acid (D), and premature stop (E) in mutant *m863*.

The mutant allele *m863* was genetically mapped to zebrafish linkage group 18 (LG18) between the SSLP markers z13220 (72.4 cM on MGH panel) and z59637 (74.0 cM). This interval was further narrowed down proximally by generating the polymorphic microsatellite marker 18s56, for which no recombinants were detected in a cross representing 2728 meioses (Fig 2A), revealing that the gene affected by *m863* must be tightly linked to this marker. Based on the Ensembl Zv8 assembly, in the critical interval *zgc*:*63840* was identified as a candidate gene for the *m863* mutant allele (Fig 2A). Sequence analysis by BLAST (Ensembl, Sanger Center) revealed *zgc*:*63840* as homolog of the DDB1 encoding gene of vertebrates, including mouse, human, rat, bovine, and chicken (S1 Fig), the invertebrate *Drosophila*, and the plant *Arabidopsis thaliana*. Hence, in the following, *zgc*:*63840* will be named *ddb1* in agreement with the current Zv9 genome annotation (www.zfin.org). The *ddb1* cDNA was cloned from *in vitro* reverse transcribed mRNA of individual 2-day-old-embryos using primer pairs (see S1 Table) designed based on the reference sequence (*zgc*: *63840*, NM_200626.1). The resulting zebrafish *ddb1* cDNA (submitted to GenBank: JQ692623.1) contained an open reading frame of 3420 bp encoding a polypeptide of 1140 amino acids, which is around 250 amino acid residues longer than the previously predicted protein product NP_956920.1 (S2 Table). The Ddb1 protein deduced from our cloned zebrafish *ddb1* (submitted to GenBank: AFI92852.1) is of the same length as previously documented DDB1 proteins from other vertebrate species, including human, mouse, rat, bovine, and chimpanzee (S1 Fig and S2 Table).

A multi-sequence alignment of vertebrate DDB1 proteins revealed that DDB1 is highly conserved at the amino acid level. Zebrafish Ddb1 has high identity (about 79% identity at DNA and 90% identity at protein level) to mammals, including human, rat, chimpanzee, bovine, and mouse DDB1. A similar percentage of DDB1 identity was also detected for the non-mammalian vertebrates chicken (81% and 91.1% at DNA and protein level, respectively). The domain analysis of the zebrafish Ddb1 confirmed the known conserved features of DDB1 protein: three β-propeller domains (BPA, BPB and BPC) and a C-terminal helical domain (CTD) (S1 Fig) [5, 8, 10, 36].

To determine whether the *m863* mutation indeed affects *ddb1*, cDNA was prepared from individual wild type and *m863* ^-/-^ 2 dpf old embryos and sequenced. In *m863* ^*-/-*^ mutant embryos, an insertion of 4 bp at the end of exon 19 (2402-2405nt of *ddb1* ORF) was detected in the *ddb1* transcript (Fig 2B). When genomic *ddb1* DNA was sequenced, a mononucleotide substitution (T→C) in the conserved GT splice donor site of *ddb1* intron 19 was detected (Fig 2C). This point mutation inactivated the correct splice donor site and caused an adjacent cryptic splice site to become active, leading to partial intron insertion into the transcript. As a result, in the *ddb1* ORF after amino acid residue 800 (Glu or E) a frameshift occurred, with a stop codon after a stretch of 55 additional non-Ddb1 amino acids (Fig 2D). The truncated *m863* Ddb1 protein lacks the C-terminal part of the wild-type sequence, corresponding to the CTD and most of the BPC propeller domain of Ddb1 protein (Fig 2E), based on comparison to human DDB1 domain structure [5, 36]. These domains have been shown to be involved in the formation and function of the BPA-BPC double-propeller fold, which is critical for DDB1-CUL4 mediated specific protein-protein interactions [30, 37, 38].

To confirm that the phenotype caused by the *m863* mutation is in the *ddb1* gene, *ddb1* morpholinos (MO) were used to phenocopy the *m863* mutant phenotype. A splice-site (*ddb1*_MO1) and a translational start-site targeted morpholino (*ddb1*_MO2) were used, and their knockdown efficiencies tested (S2 Fig). The splice site MO *ddb1*_MO1 would only affect zygotic expression of *ddb1*, while the ATG-morpholino *ddb1*_MO2 would potentially affect translation of both maternal and zygotic transcripts. To test the efficiency of the *ddb1*_MO2, it was co-injected with a plasmid expressing the *ddb1* 5' UTR and ATG with morpholino target site linked to the EGFP ORF, using the CMV promoter. *ddb1*_MO2 mediated elimination of plasmid derived EGFP in 72 hpf larvae (S2D Fig), revealing that the morpholino effectively blocked Ddb1 translation even on the third day of development. Both *ddb1*_MO1 and *ddb1*_MO2 mediated knockdown of Ddb1 effectively induced a phenotype with reduced number of *th*-expressing DA neurons in the pretectum and retina that was similar to the *m863* mutant phenotype (Fig 1A–1F; S3A–S3F Fig; Table 1, and unpublished data). To evaluate whether *ddb1*_MOs may cause *p53*-dependent off-target effects [39], we co-injected *p53*_MO into one-cell stage embryos and analyzed *th* expression at 3 dpf. *ddb1*_MO and *p53*_MO coinjected embryos reduced *th* signal in pretectum and retina similar to *ddb1* morphants (S3D–S3F and S3G–S3I Fig). Thus co-injection of *p53*_MO did not attenuate the DA neuron phenotype of the morphants (S3D–S3I Fig and Table 1), suggesting that the morphant phenotype was not caused by non-specific morpholino induced p53 dependent cell death [39]. The fact that the *ddb1*_MO2, while potentially eliminating translation from maternal and zygotic messages, did not induce a phenotype significantly stronger than the zygotic mutant or *ddb1*_MO1 morphant phenotypes may argue that maternal rescue may not solely derive on maternally deposited *ddb1* mRNA (S4 Fig and text below), but also on maternally derived Ddb1 protein deposited in the oocyte. We attempted to generate maternal and zygotic mutant embryos by plasmid transgene rescue of *ddb1*, but did not succeed (unpublished data—we think that a specific amount of Ddb1 may be needed to sustain proper development). Therefore, we cannot evaluate a potential complete loss of Ddb1 activity, both maternal and zygotic, in zebrafish.

**Table 1:** Dopaminergic phenotype in *ddb1*_MO and *p53*_MO knockdown larvae.

	Morpholinos		Embryos	Phenotypic class				
	*ddb1* MOs	*p53*_MO	Total No.	I	II	IIIa	IIIb	IV
***ddb1*_MO1**	4ng	0ng	39	8	7	10	14	0
	4ng	4ng	62	7	13	17	25	0
	6ng	0ng	16	1	0	3	12	0
	6ng	6ng	51	1	5	25	17	3
	8ng	0ng	39	0	1	0	38	0
	8ng	7ng	28	0	0	10	18	0
***ddb1*_MO2**	6ng	0ng	29	3	12	14	0	0
	6ng	6ng	31	2	14	15	0	0
	8ng	0ng	25	0	0	4	19	2
	8ng	7ng	38	3	0	11	21	3

Summary of results from *ddb1* morpholino knockdown experiments. Amounts of morpholino injected per embryo are given in ng. Injected embryos were allowed to develop until 72 hpf, fixed, and stained by WISH for *th* expression. Zebrafish larvae from each knockdown experiment were classified into different phenotypic classes according to the effect of the knockdown on the *th* expressing pattern and on morphology. I, *th* expression pattern was similar to wild type siblings; II, *th* expressing cells were slightly reduced in number; IIIa, *th* expressing cell number was reduced in the pretectum and retina; IIIb, *th*-expressing cell number clearly reduced plus decreased size of brain and/or retina; IV, dying and decaying embryos. Based on the changes in *th*-expression pattern, the class IIIa/b phenotype was similar to that of *m863* mutants.

### *ddb1* is expressed broadly in early development and enhanced in proliferation zones

**Fig 3:**
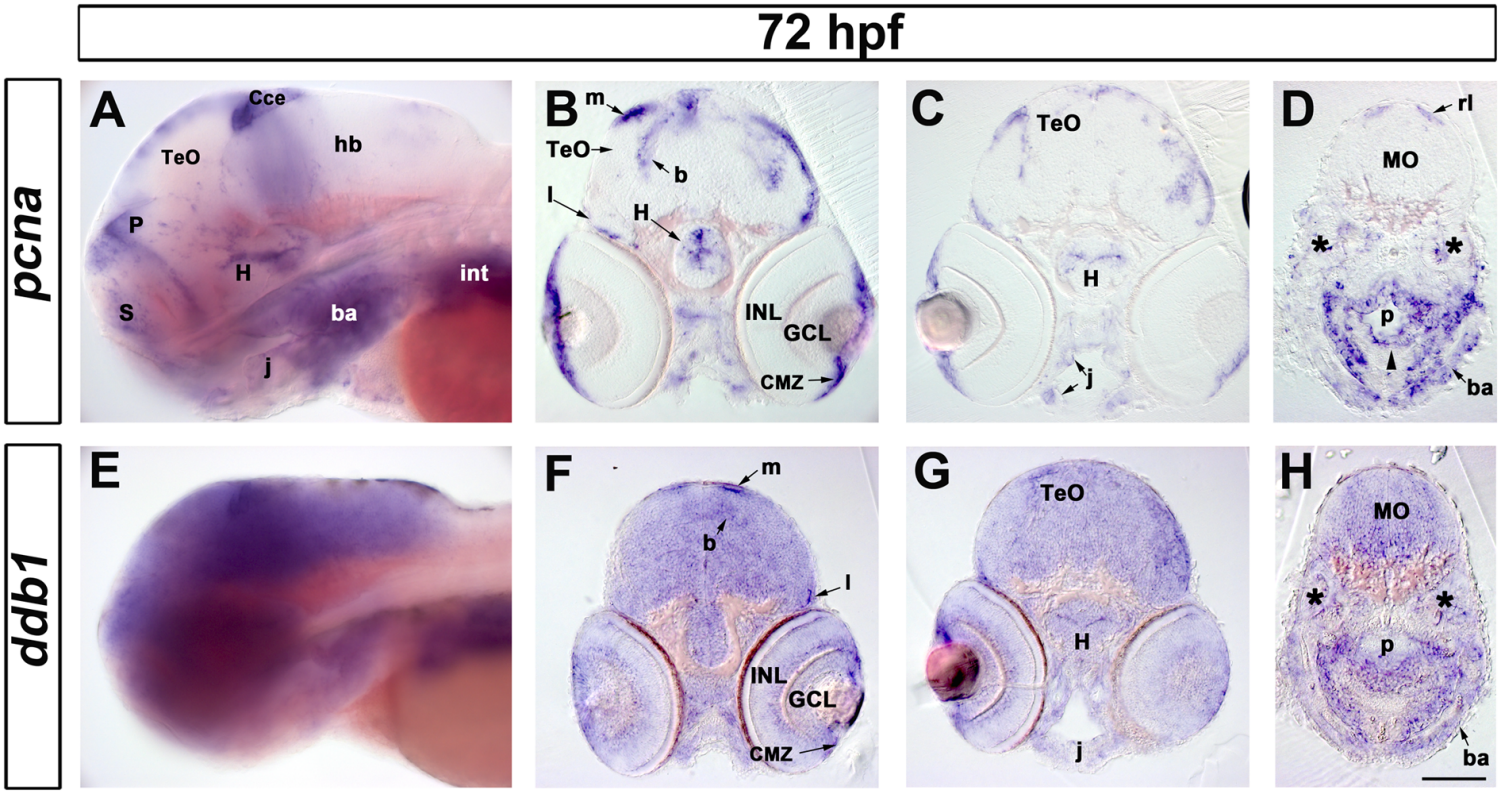
Expression of *ddb1* in proliferation regions in wild type larvae at 72 hpf. (A-H) Characterization of *pcna* (A-D) and *ddb1* (E-H) expression in wild type larvae at 72 hpf. Lateral views (A, E) and sections (20 μm; B-D, F-H). (A, E) *ddb1* was broadly expressed in the brain while *pcna* was restricted to proliferation zones. (B-D, F-H) In the medial (m), lateral (l) and basal (b) proliferation zones of the tectum opticum (TeO) and the hypothalamic proliferation zone (H), elevated expression of *ddb1* was detected compared to other areas of the brain. *ddb1* was also highly expressed in the ciliary marginal zone (CMZ) and in proliferation regions of the pharyngeal endoderm (p) (black arrow head), jaw (j) and branchial arches (ba). The expression of *ddb1* was also found in *pcna*-negative regions such as the ganglion cell layer (GCL) and inner nuclear layer (INL) of the retina. Transcripts of *ddb1* were detected throughout the medullar oblongata (MO) where *pcna* was only expressed in the dorsal part, the rhombic lip (rl). Anterior towards the left. Stars mark the otic vesicle. Scale bar: 100 μm.

Expression analysis by whole mount in situ hybridization (WISH) revealed that *ddb1* mRNA was maternally deposited in the oocyte and detectable from the one cell stage on (S4A–S4F and S4S Fig). It continued to be broadly expressed zygotically in the embryo, but appeared at elevated levels in proliferation regions in larvae at 72 hpf, including the ciliary marginal zone of the retina and the ventricular surfaces of the brain (Fig 3E–3G and S4J–S4R Fig). We performed Vibratome sections from WISH embryos marked by riboprobes for either *ddb1* or *proliferating cell nuclear antigen* (*pcna*), which encodes a non-histone nuclear protein frequently used as a cell proliferation marker [40]. The results revealed that *ddb1* transcripts were detected in both proliferating and non-proliferating tissues in wild type larvae at 72 hpf (Fig 3A and 3E). Although ubiquitously expressed in the brain, *ddb1* transcripts were detected at elevated levels in proliferative regions where *pcna* was also expressed. Regions with enhanced *ddb1* WISH signal in the lateral and basal proliferation zone of the optic tectum (Fig 3A–3C and 3E–3G), hypothalamus (Fig 3A–3C and 3E–3G), retina ciliary marginal zone (CMZ) (Fig 3B, 3C, 3F and 3G) were also marked by the expression of *pcna*. In non-neural tissues such as the pharyngeal endoderm, jaw, and branchial arches, *ddb1* expression was also enhanced in regions of *pcna* expression (Fig 3A, 3C, 3D and 3G–3H).

We also determined *ddb1* expression in *ddb1* ^*m863*^ mutant embryos by WISH and semi-quantitative RT-PCR (S5 Fig). The *ddb1* WISH signal was strongly reduced in 24 hpf *ddb1* ^*m863*^ mutant embryos, and essentially absent from 72 hpf mutant larvae (S5A–S5I Fig). As the WISH probe efficiency should not be affected by the mutation only at the intron 19 splice donor site, this finding indicates that the aberrantly spliced *ddb1* ^*m863*^ mutant mRNA may be highly instable. We confirmed this finding by *ddb1* RT-PCR on wild type, heterozygous, and homozygous mutant *m863* siblings and quantification of the PCR bands (S5J and S5K Fig). If the loss of signal is caused by instability of the mutant transcript, the *ddb1* residual signal at 24 hpf may represent the maternal transcript. However, we can also not exclude that Ddb1 activity may be required to maintain *ddb1* expression.

### Reduction of proliferating cells and enhanced apoptosis in *ddb1* ^*m863*^ mutants

*ddb1* ^*m863*^ zygotic mutant embryos showed an increase of cell death from the second day of development onwards and developed smaller brain and eyes at 72 hpf when compared to wild-type siblings. Given that *ddb1* transcription is elevated in brain proliferation zones, we wanted to know whether the reduced size of brain and eyes may be due to activation of programmed cell death throughout the brain, or selectively in the proliferation regions. Therefore, we performed *pcna* WISH and TUNEL assays in *ddb1* ^*m863*^ mutants at different developmental stages.

**Fig 4:**
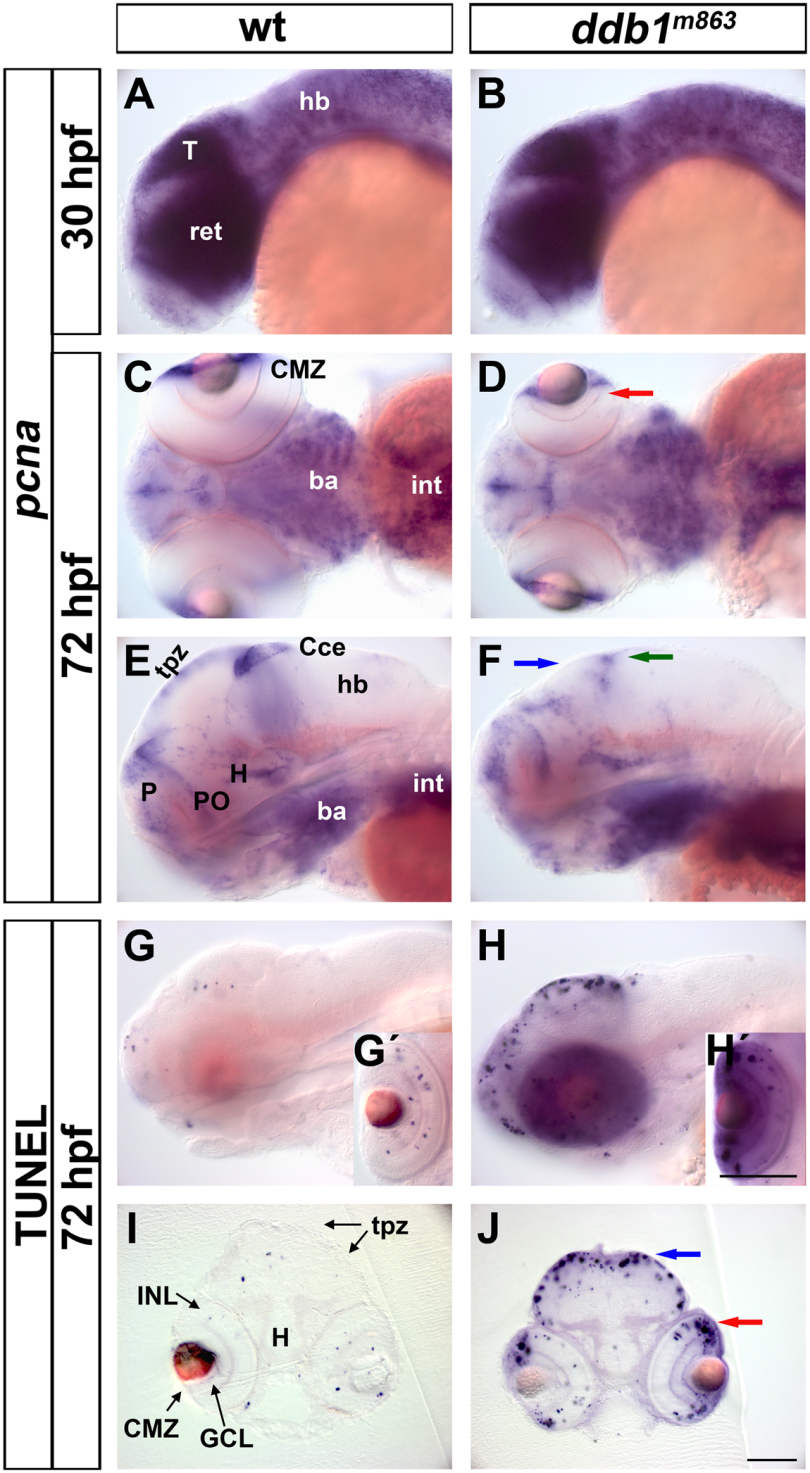
Enhanced apoptosis in proliferation regions of the *ddb1* ^*m863*^ mutant CNS. (A-F) The *pcna* expression pattern in *ddb1* ^*m863*^ mutants and wild type siblings at 30 hpf (A-B) and 72 hpf (C-F). (G-J) TUNEL assay for apoptosis in *ddb1* ^*m863*^ mutants (H-H', J) and wild type siblings (G-G', I) at 72 hpf. Embryos or larvae in lateral (A-B, E-H) and ventral views (C-D). Cross sections (20 μm; I-J) from larvae by TUNEL assay. Arrows represent affected *pcna*-expressing cells or enhanced apoptotic cells in the retina (D, J; red), pretectal region (F, J; blue), and cerebellum (F; green). Abbreviations used: ba, branchial arches; CMZ, ciliary marginal zone; Cce, cerebellum; GCL, ganglion cell layer; hb, hindbrain; H, hypothalamus; INL, inner nuclear layer; int, intestine; P, pallial proliferation zone; PO, preoptic area; tpz, tectal proliferation zones; Anterior towards the left. Scale bars in H' for G' and H', in J for all of others: 100 μm.

The WISH assay revealed that *pcna* expression was differentially affected in homozygous *ddb1* ^*m863*^ mutants during development. At 30 hpf, no obvious differences of *pcna* expression were detected between homozygous *ddb1* ^*m863*^ mutants and their wild type siblings (Fig 4A and 4B). At 72 hpf, however, the expression pattern of *pcna* was selectively affected in various brain proliferation zones of *ddb1* ^*m863*^ mutant embryos when compared to wild type siblings. *pcna* expression was slightly diminished and appeared in a more diffuse way in the retinal CMZ (Fig 4C and 4D), whereas it was severely reduced in the tectal proliferation zones (tpz) and cerebellum of homozygous *ddb1* ^*m863*^ mutants in comparison to wild type siblings (Fig 4E and 4F). No obvious alterations of *pcna* expression were found in other proliferation regions of *ddb1* ^*m863*^ mutant brain, including the pallial proliferation region, ventricular zone and hypothalamus (Fig 4C–4F). One possible explanation for the differential effect on neural structures may be that persistent maternally derived functional Ddb1 protein may rescues the zygotic *ddb1* mutant phenotype of early proliferating brain regions, while late proliferating regions depend on zygotically expressed *ddb1*.

The reduction of *pcna* expression in the proliferation regions of the tectum, cerebellum, and retina was reminiscent of the apoptotic pattern of *ddb1* ^*m863*^ mutants. TUNEL assay showed that at 72 hpf there were more apoptotic cells in the retinal CMZ, tpz, and cerebellum than in other regions of the brain in homozygous *ddb1* ^*m863*^ mutants compared to wild type siblings (Fig 4G and 4H'). Sections of these TUNEL assay embryos confirmed that the programmed cell death was located prominently in the *pcna*-expressing tpz and retinal CMZ in *ddb1* ^*m863*^ mutants (Fig 4I and 4J).

### Transcriptional activation of *p53* in *ddb1* ^*m863*^ mutants

To investigate whether proliferating cells in *ddb1* ^*m863*^ mutant embryos are selectively eliminated by apoptosis, we analyzed the expression of *p53* (*tp53*) [41] in *ddb1* ^*m863*^ mutants during development by WISH. *p53* encodes the tumor suppressor protein p53, a transcription factor involved in control of various cellular programs, including cell cycle arrest, apoptosis, DNA repair, and cellular senescence [42].

**Fig 5:**
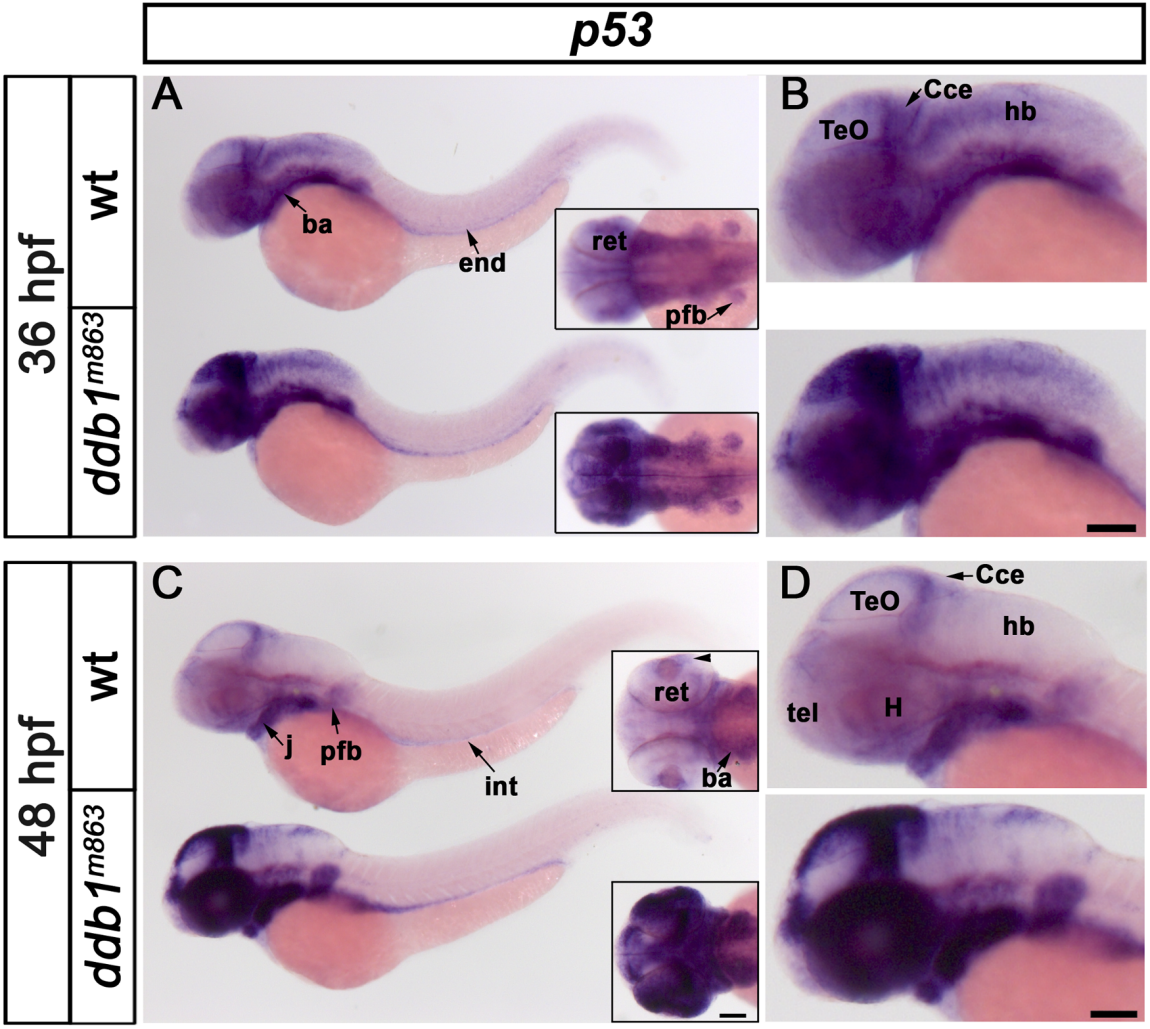
Increase of *p53* transcript levels in *ddb1* ^*m863*^ mutants. (A-D) Lateral (A-D) and dorsal views (inserts in A, C) of *p53* expression pattern in embryos at 36 hpf (wt, n = 30; mut, n = 8) and 48 hpf (wt, n = 25; mut, n = 11). The transcription of *p53* was prominently enhanced in *ddb1* ^*m863*^ mutant embryos. Anterior towards the left. Abbreviations used: ba, branchial arches; Cce, cerebellum; end, endoderm, H, hypothalamus, hb, hindbrain; j, jaw; pfb, pectoral fin bud; tel, telencephalon; TeO, tectum opticum. Scale bar: 100 μm

In 36 hpf wild type embryos, *p53* was expressed in the telencephalon, retina, midbrain, cerebellum, hindbrain, otic capsule, branchial arches, pectoral fin bud, and endoderm (Fig 5A and 5B, top). At 48 hpf, the expression level of *p53* has declined and was restricted spatially to the proliferation regions, including optic tectum, retina, ventricle zone, branchial arches and other non-brain tissues (Fig 5C and 5D, top). In homozygous *ddb1* ^*m863*^ mutants at 36 hpf, *p53* expression levels were strongly increased in the tpz, MHB, cerebellum, and retina, moderately in the ventricular zone, and branchial arches, and slightly in the hindbrain, pectoral fin bud, and endoderm (Fig 5A and 5B, bottom). *p53* transcripts were even more severely enhanced in *ddb1* ^*m863*^ mutant embryos at 48 hpf. The increase of *p53* transcription in *m863* mutants was most prominent throughout the retina, in the dorsal diencephalon, tpz, MHB, cerebellum, pectoral fin bud, and branchial arches, and was moderate in the hindbrain and endoderm when compared to wild type siblings (Fig 5C and 5D). These data suggest that transcription of the tumor suppressor gene *p53* was enhanced in *ddb1* ^*m863*^ mutant embryos, with proliferation regions most severely affected.

### Inactivation of p53 leads to rescue of the ddb1m863 mutant apoptosis phenotype

To determine whether the accumulation of *p53* was responsible for the enhanced apoptosis in *ddb1* ^*m863*^ mutants, we inactivated *p53* expression by morpholino knockdown in *ddb1* ^*m863*^ mutants. *p53* morpholino was microinjected at several concentrations into one cell stage embryos from intercrosses of heterozygous *ddb1* ^*m863*^ parents: group A (non-injected), group B (6 ng *p53* MO per embryo) and group C (12 ng *p53* MO per embryo). The injected embryos were fixed at 72 hpf, analyzed by TUNEL assay, and genotyped by PCR.

**Fig 6:**
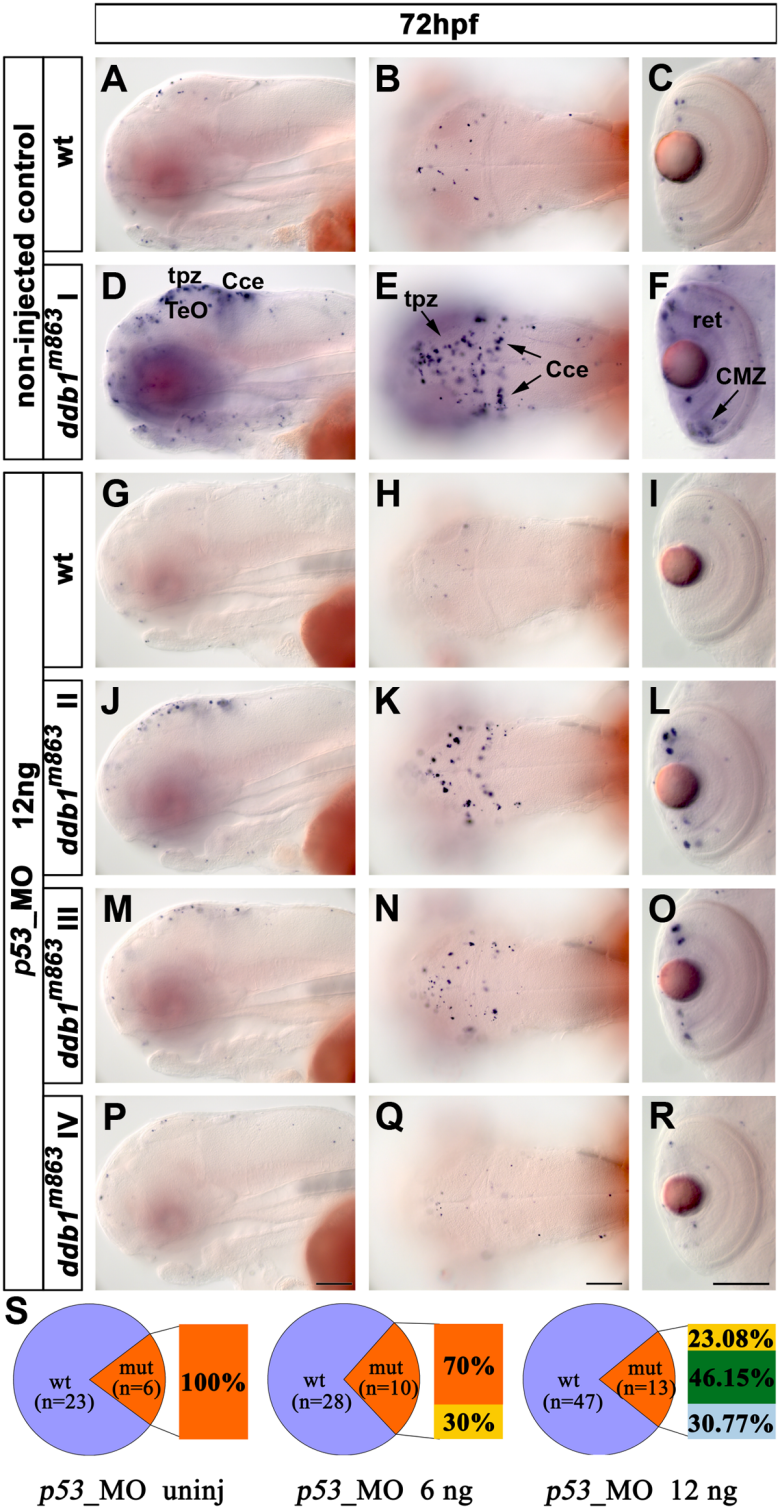
Knock down of *p53* rescued the apoptosis phenotype of *ddb1* ^*m863*^ mutants. (A-F) Enhanced apoptosis in *ddb1* ^*m863*^ (class I phenotype; D-F) compared to wild type siblings (A-C) without *p53*_MO injection. (G-R) Partial (class II and class III; J-O) and complete (type IV; P-R) rescue of *ddb1* ^*m863*^ apoptosis phenotype compared to wild siblings (G-I) after injection of 12 ng *p53*_MO per embryo. (S) Percentage distribution of mutant and rescued mutant phenotypes. Genotypes were determined by PCR. Class I (orange), mutants showed the severest apoptosis phenotype; class IV (light blue) mutants have the weakest phenotype with only a few of apoptotic cells, similar to their wild type siblings; class II (yellow) and III (green) are intermediate phenotypes. Abbreviations used: tpz, tectal proliferation zone; Cce, cerebellum; ret, retina; Anterior towards the left. Scale bar: 100 μm.

Based on the TUNEL assay results, the phenotypes of the PCR genotyped *ddb1* ^*m863*^ mutant embryos were classified into four classes according to the number of apoptotic cells observed. Based on PCR genotypes, genetically mutant embryos were 20.7% for group A (n = 29), 26.3% for group B (n = 38) and 21.7% for group C (n = 60) (Table 2). At 72 hpf, apoptosis was prominently elevated in the tpz, retinal CMZ and cerebellum in non-injected homozygous *ddb1* ^*m863*^ mutants (class I phenotype) when compared to wild type siblings (group A) (Fig 6A–6F). When *p53*_MO was injected at an amount of 6 ng per embryo (group B), 30% (n = 10) of mutant embryos showed less apoptotic cells (class II), whereas 70% (n = 10) of them were similar to the mutant phenotype in group A (non-injected) (Fig 6S and Table 2). When 12 ng *p53*_MO per embryo was injected (group C), the apoptosis in mutant embryos was greatly alleviated compared to group A and group B (Fig 6A–6S and Table 2). In group C, 23.08% (n = 13) of mutant larvae showed reduced numbers of apoptotic cells (class II) (Fig 6J–6L and 6S, Table 2), and 46.15% (n = 13) of the mutants showed strongly reduced numbers of TUNEL stained cells (class III) (Fig 6M–6O and 6S; Table 2). In addition, 30.77% of mutant larvae (class IV, n = 13) in group C showed only very few apoptotic cells and were indistinguishable from wild type siblings (Fig 6G–6I and 6P–6S; Table 2). These data demonstrated that inactivation of *p53* rescued apoptosis in *ddb1* ^*m863*^ mutants in a dosage dependent manner, revealing that the apoptotic phenotype of *ddb1* ^*m863*^ mutants depends on *p53* activity.

**Table 2:** Knockdown of *p53* by anti-sense morpholino compensates the apoptotic phenotype of *ddb1* ^*m863*^ mutants.

	Morpholino	Embryos Total	Embryos wildtype	No. of embryos per class				
Group	*p53*_MO	No.	No.	total	class I	class II	class III	class IV
A	0ng	29	23	6	6	0	0	0
B	6ng	38	28	10	7	3	0	0
C	12ng	60	47	13	0	3	6	4

The *ddb1* ^*m863*^ mutants at one-cell stage were injected with *p53* morpholino as indicated (amount of morpholino per embryo provided in ng), and analyzed by TUNEL staining at 72 hpf. Based on the TUNEL staining, genotyped mutant larvae were categorized in the following classes: Class I—severe increase in apoptosis; Class IV weak phenotype with only few apoptotic cells similar to wild type siblings. The apoptosis levels in class II and III were between class I and IV (see Fig 6).

### Transcriptional deregulation of cell cycle genes in *ddb1* ^*m863*^ mutant embryos

Several cell cycle regulators, including the cyclin-dependent kinase inhibitors p21^CIP1/WAF1^ (Cdkn1a) and p27^KIP1^ (Cdkn1b), and the cyclin CcnE, are known to be targeted for degradation by the DDB1-CUL4A ubiquitin ligase [7]. In mice with conditional DDB1 knockout in the CNS, the protein levels of p21^CIP1/WAF^ and p27^KIP1^ are increased while cyclin E and A are unaltered [25]. However, whether the depletion of *ddb1* function may alter the transcription of cell cycle genes is unclear. Therefore, we compared expression of the two paralogues of *p21* ^CIP1/WAF1^ (*p21* ^*CIP1/WAF1*^ *a* and *b*) and *p27* ^*KIP1*^ (*p27* ^*KIP1*^ *a* and *b*), as well as of *p57* ^*KIP2*^ (*cdkn1c)*, *ccna2*, *ccnd1*, and *ccne2* in *ddb1* ^*m863*^ mutants and wild type siblings by WISH. Mutant and wildtype siblings were stained in one batch for each probe and documented photographically. The genotype of each embryo was determined by PCR using genomic DNA extracted from the fixed and stained material.

**Fig 7:**
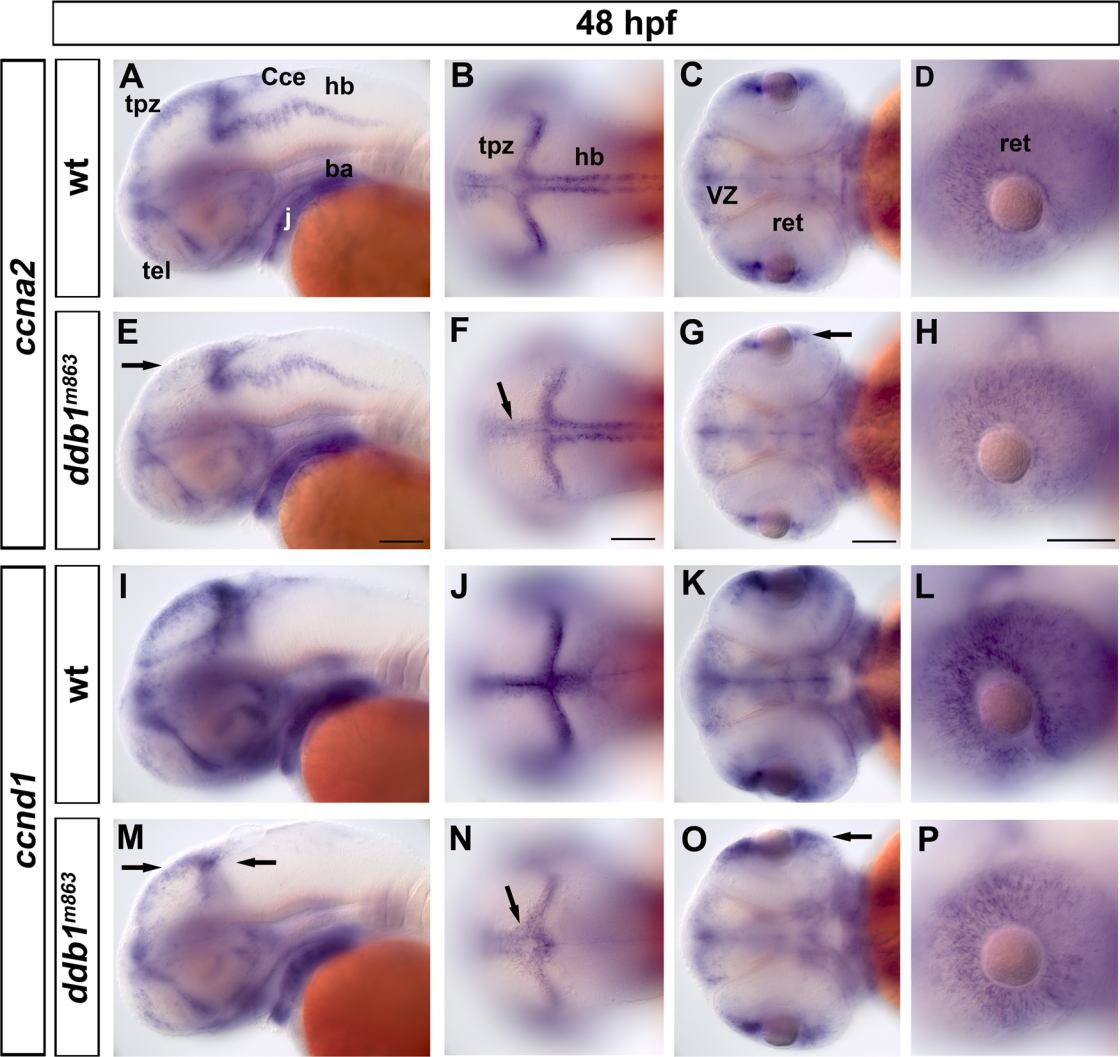
Reduced *ccna2* and *ccnd1* expression in *ddb1* ^*m863*^ mutant embryos. (A-D) Expression pattern of *ccna2* in wild type siblings. (E-H) Slight reduction of *ccna2* expression was detected in the tpz (arrow in E-F) and CMZ (arrow in G) in *ddb1* ^*m863*^ mutants. (I-P) The expression pattern of *ccnd1* in wild type and homozygous *ddb1* ^*m863*^ embryos. The transcripts of *ccnd1* were slightly decreased in homozygous mutants (M-P). Arrows represent the altered expression of *ccnd1* in the tectal proliferation region (M, N), cerebellum (M), and retina (O, P). Abbreviations used: ba, branchial arches; Cce, cerebellum; j, jaw; hb, hindbrain; tel, telencephalon; ret, retina; tpz, tectal proliferation region; VZ, ventricular zone. Anterior towards the left. Scale bars: 100 μm.

The results showed that there were no obvious alterations in WISH signal intensities of *p27* ^*KIP1*^, *p57* ^*KIP2*^ and *ccne2* in *ddb1* ^*m863*^ mutants (unpublished data), while *ccna2* and *ccnd1* WISH signal was significantly reduced (Fig 7). For *ccna2*, reduced signals were mainly observed in the tectal proliferation zone and CMZ in homozygous *ddb1* ^*m863*^ mutants, whereas WISH signal intensity in hindbrain proliferation zone and outside the brain appeared to be similar to wild type siblings (Fig 7A–7H). Similarly, a reduction in *ccnd1* WISH signal was observed in *ddb1* ^*m863*^ mutant embryos in the CMZ and the tectal, cerebellar and ventricular proliferation zones (Fig 7I–7P).

**Fig 8:**
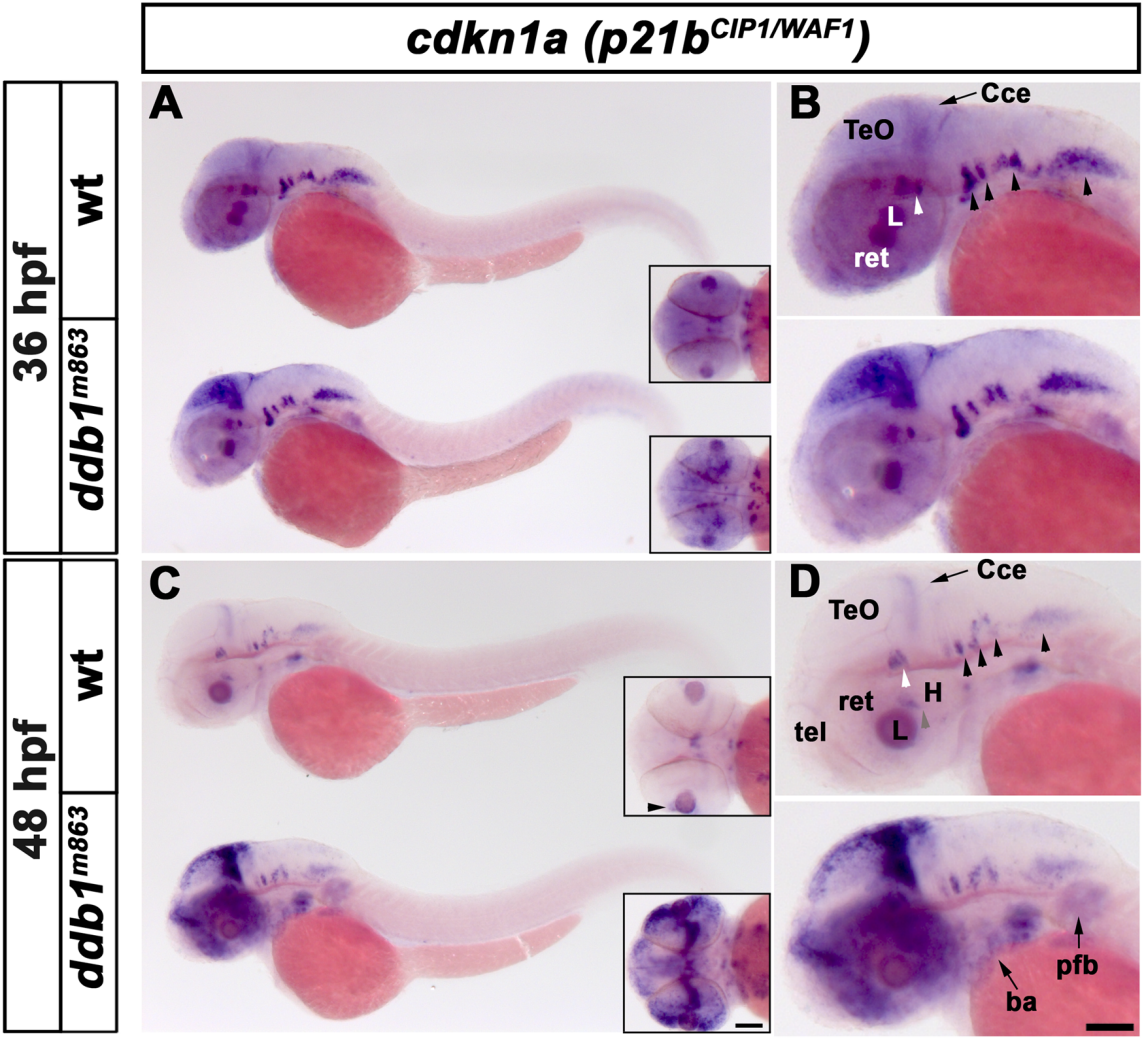
Increased *p21b* ^*CIP1/WAF1*^ expression in homozygous *ddb1* ^*m863*^ mutants. (A-B) Enhanced expression of *p21b* ^*CIP1/WAF1*^ (*cdkn1a*) in *ddb1* ^*m863*^ homozygous embryos in the proliferation regions of the optic tectum, compared to wild type siblings at 36 hpf. (C-D) At 48 hpf, *p21b* ^*CIP1/WAF1*^ expression was strongly elevated in proliferation regions of the optic tectum, cerebellum, telencephalon, retina, branchial arches and pectoral fin bud in *ddb1* ^*m863*^ mutants compared to wild type siblings. In contrast, expression of *p21b* ^*CIP1/WAF1*^ in ventral hindbrain nuclei (arrow heads in B, D) was unaltered in *ddb1* ^*m863*^ mutants. All views are lateral except for inserts (dorsal views). Abbreviations used: Cce, cerebellum; H, hypothalamus; L, lens; pfb, pectal fin bud; ret, retina; tel, telencephalon; TeO, tectum opticum. Anterior towards the left. Scale bar: 100 μm.

In contrast, *p21* ^*CIP1/WAF1*^ WISH signal intensity strongly increased in *ddb1* ^*m863*^ mutants (Fig 8). When analyzing the expression of the two zebrafish paralogues of *p21* ^*CIP1/WAF1*^, we found that the expression pattern of *p21a* ^*CIP1/WAF1*^ was broader than that of *p21b* ^*CIP1/WAF1*^, which appeared in a more spatial-restricted pattern (Fig 8 and unpublished data). To determine potential effects of Ddb1 on *p21* ^*CIP1/WAF1*^ spatial expression, we focused our analysis on expression of *p21b* ^*CIP1/WAF1*^. In 36-hpf wild type embryos, *p21b* ^CIP1/WAF1^ was highly expressed in several ventral cell groups in the hindbrain and ventral midbrain and in the lens, while weak expression was observed in other brain regions and pectoral fin buds (Fig 8A and 8B). In 36 hpf homozygous *ddb1* ^*m863*^ mutants, however, the expression of *p21b* ^*CIP1/WAF1*^ was increased strongly in the dorsal midbrain and slightly in pectoral fin buds, while its WISH signal intensities in other regions were similar to wild type siblings (Fig 8A and 8B). In 48-hpf wild type embryos, *p21b* ^*CIP1/WAF1*^ expression in most regions was weaker compared to 36 hpf, and hardly detectable in the forebrain and pectoral fin bud, whereas weak expression persisted in the eyes, ventral midbrain, mid-hindbrain boundary (MHB), hindbrain nuclei, and developing branchial arches (Fig 8C and 8D). In contrast, in 48 hpf *ddb1* ^*m863*^ mutants *p21b* ^*CIP1/WAF1*^ expression were strongly increased in the ventricular zone, the tpz, MHB, cerebellum, and retina, as well as elevated moderately in the pectoral fin bud and branchial arches (Fig 8C and 8D).

## Discussion

We analyzed the zebrafish homologue of the human *DDB1* gene for its activities during development. DDB1 has been demonstrated to contribute to several essential cellular mechanisms, including DNA-damage repair, cell proliferation, survival, and genomic stability [2–5]. DDB1 is also involved in formation of CUL4-RING E3 ligase complexes, which are associated with targeting and proteolysis of specific substrates protein using the ubiquitin-dependent pathway [7–9]. DDB1 is a large multidomain CUL4-adaptor protein containing three β-propellers (BPA, BPB, and BPC) and a CTD domain [36]. The BPB domain mediates predominantly the formation of the DDB1-CUL4 complex, whereas a large clam-shaped double β-propeller pocket BPA-BPC is associated with substrate protein recruitment [8, 10, 25]. Zebrafish Ddb1 shares high sequence identity with its orthologs in mammalian and non-mammalian vertebrates (about 90% protein sequence identity to human, rat, chimpanzee, bovine, mouse, chicken, and *Xenopus* DDB1), invertebrates (*Drosophila* 61%) and plant species (*Arabidopsis* 54%). The high sequence conservation also suggests a strong functional conservation.

### Ddb1 in zebrafish development

We identified a new *ddb1* ^*m863*^ mutant allele from a chemical ENU mutagenesis screen, and analyzed its functions in zebrafish early development. The mutation in the *ddb1* ^*m863*^ allele affects proper splicing of exon 19 to exon 20, causing Ddb1 protein to be truncated after amino acid 800, lacking half of the BPC and the whole CTD conserved domains. *ddb1* ^*m863*^ zygotic mutant embryos and early larvae were morphologically similar to wildtype siblings on the first and second day. On the third day, however, when organogenesis and growth from local proliferation zones prevail, a prominent pleiotropic phenotype with reduced size and abnormal structure of the eyes, brain, and head skeleton can be observed. To understand the function of *ddb1* in zebrafish, we analyzed its expression pattern throughout embryonic and early larval stages. Both RT-PCR and whole mount *in situ* hybridization revealed that *ddb1* was maternally expressed and mRNA deposited in oocytes. Thus, maternally derived Ddb1 protein may compensate Ddb1 loss in *ddb1* ^*m863*^ mutants during early developmental stages and rescue potential developmental defects through cleavage, gastrulation and somitogenesis stages.

Similar to the expression profiles in mouse [25], *ddb1* mRNA was ubiquitously detected in early zebrafish embryos. In embryos at post-somitogenesis stages during the third day of development, however, *ddb1* expression became more restricted to the CNS, head skeleton, pharyngeal region, and endoderm. We found that areas with elevated *ddb1* transcription levels were correlated with *pcna* expression and therefore with regions of cell proliferation. When analyzing the larval brain at 3 dpf, this correlation was most pronounced in the proliferation zones in the tectum, the cerebellum, the subventricular zone, and the CMZ of the retina. The zebrafish CNS and retina grow over its lifetime, and new cells are constantly added from proliferation zones [43, 44]. The non-proliferation zone areas of the brain and retina consist largely of differentiated cells, which have lost their proliferating capacity. Hence, we presume that cells with a continued potential to proliferate may rely on high levels of *ddb1* expression.

In other animal model systems, including *Drosophila melanogaster* [32, 33, 45], and mouse [25, 46], deficiency of DDB1 leads to early embryonic lethality, while tissue specific knockouts affect proliferation and development in the studied tissues. In zebrafish, recent studies revealed that the malformation of pectoral fins and otic vesicle after treatment with the drug thalidomide are caused by its binding to Crbn, a substrate receptor of DDB1-CUL4 E3 complexes, and inhibition of CUL4 E3 ubiquitin ligase activity [47]. The resulting aberrant accumulation of DDB1-CUL4 ligase substrates and abnormal proliferation and differentiation by deregulated FGF8, at least partially, have been implied to be responsible for the thalidomide developmental defects. The importance of CUL4 E3 system in fin development is further supported by data from zebrafish *cul4a* morphants [48]. In contrast to these publications, our data did not reveal morphologic fin defects in *ddb1* ^m863^ mutants. However, our data showed that the brain and eyes are affected in *ddb1* ^m863^ mutants, which have been reported to develop normally in *zcrbn* and *cul4a* morphants [47]. Interestingly, recent data indicate increased apoptosis and decreased PCNA-positive proliferating cells in *cul4a* morphants [48], which is similar to our observations of *ddb1* ^m863^ mutants, albeit studies focused on different tissues. It appears that the DDB1-CUL4 E3 complex has important functions in cellular apoptosis and proliferation. Given the reported differences in PCNA expression in different tissues, additional tissue-specific factors are likely involved. This is in line with DDB1-CUL4 E3 complexes containing basic DDB1-CUL4-ring plus various substrate receptors, and affecting diverse downstream targets. Such tissue selectivity has been reported with *fgf8* as target of CUL4-CRBN in fins [47], and *tbx5* as target of Cul4a in cardiovascular development [48]. Thus, the discrepancy between phenotypes reported could be caused by combination of DDB1-CUL4-Ring with tissue specific substrate receptors and downstream targets [47]. Given that the *ddb1* ^m863^ mutant phenotype is again different from CRBN or CUL4, we speculate that distinct pathways with distinct substrate receptors and targets may be associated with DDB1 function in brain development.

### Ddb1 and apoptosis

We observed enhanced apoptosis in zebrafish *ddb1* ^*m863*^ mutants, most pronounced in active proliferation zones within the brain. We also observed a dramatic upregulation of *p53* transcript levels in *ddb1* ^*m863*^ mutant zebrafish. *p53* encodes the tumor suppressor protein p53 which is activated by myriad stressors such as DNA damage and oxidative stress [49, 50] and its activation can lead to apoptosis [51]. Our data revealed that enhanced expression of *p53* predominantly occurred in the proliferation zones of *ddb1* ^*m863*^ mutant embryos. Morpholino-mediated knockdown of *p53* in zebrafish *ddb1* ^*m863*^ mutants rescued the apoptosis phenotype, implying that p53 mediates apoptosis in Ddb1-deficient proliferating cells. Our findings that *ddb1* ^*m863*^ mutant larvae displayed an increase in p53-mediated apoptosis in conjunction with the specific reduction of *pcna* expression in proliferation zones prompt the notion that proliferating cells require *ddb1* function. This is in line with the data from conditional deletion of *DDB1* in the CNS and retina of mice [25], demonstrating that loss of DDB1 in the mouse brain causes p53-dependent elimination of proliferating cells. PCNA may not only act as a marker for this process, but may be directly involved, as DDB1-CUL4A has been shown to target destruction of Cdt1, a DNA replication licensing factor, only when bound to PCNA [52]. DDB1-CUL4A mediated degradation and cycling of Cdt1 has also been shown to be conserved in zebrafish [53].

The zebrafish *ddb1* ^*m863*^ mutant was initially identified by its phenotype of loss of specific dopaminergic neural groups in early larval zebrafish. Previous studies have shown that oxidative DNA damage accumulation is induced in mutant mice lacking DDB1 function in the brain and retina [25]. Similar mechanisms have been previously associated with neurodegenerative disease including Alzheimer's disease (AD) [54], PD and Amyotrophic Lateral Sclerosis (ALS) [55]. However, other types of DNA damage may occur in our *ddb1* mutants. In addition, an impairment of the ubiquitin-proteasome system (UPS), which leads to accumulation and aggregation of abnormal proteins may be a common mechanism in neurodegenerative disorders [56, 57]. In *ddb1* ^*m863*^ mutant embryos, however, we could not detect dopaminergic neurons that would have initially developed and were then lost by apoptosis. Thus, the most likely explanation for the loss of specific late differentiating dopaminergic groups in *ddb1* ^*m863*^ mutant embryos may be that programmed cell death of proliferating cells may have eliminated the neural precursor pools that give rise to these dopaminergic groups.

### Zebrafish Ddb1 and cell cycle regulators

**Fig 9:**
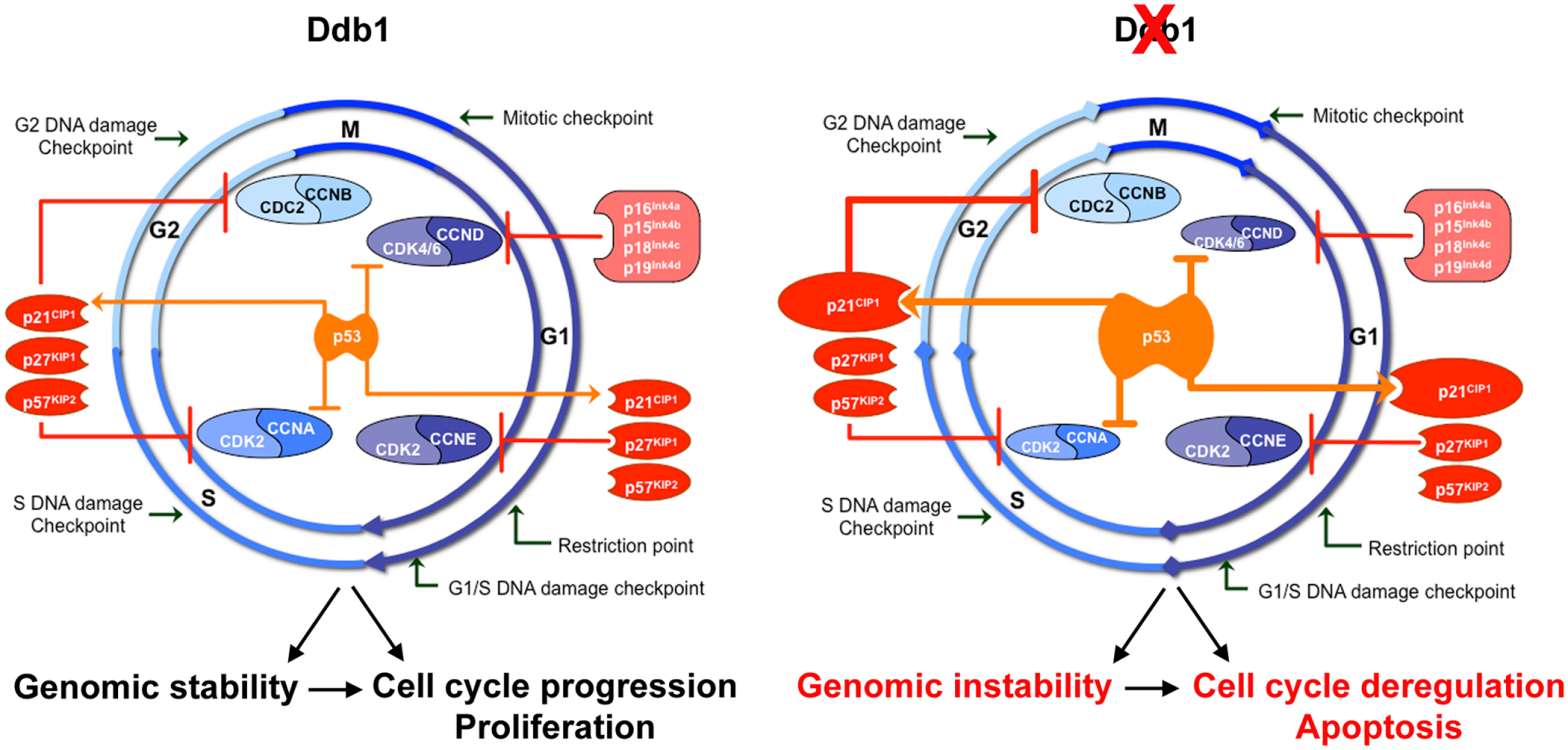
Model for DDB1 effects on regulators of cell cycle, cell survival and proliferation. Ddb1, as a component of the CUL4-DDB1 ubiquitin ligase complex, may control, among other targets and potentially in an indirect way, the expression and activity of *p53 (tp53)*. Loss of DDB1 results in enhanced p53 activity. This in turn may cause upregulation of *p21* ^CIP1/WAF1^ (*cdkn1a)*, which again contributes to downregulation of *ccnd* and *ccna* in proliferating cells. As a result, the cell cycle is deregulated and the equilibrium shifted from proliferation to apoptosis. Whether DDB1 deficiency contributes to genome instability has not been analyzed in our study.

The proper progression through each phase of the cell cycle is monitored by specific checkpoints [58, 59]. A large regulatory network controlling synthesis, phosphorylation status, and stability of checkpoint regulators guarantees proper cell cycle progression (Fig 9), and responds to incomplete cell cycle events, damaged DNA, or unfavorable extracellular environment [60]. The DDB1-CUL4 E3 ligase participates in cell cycle control by destruction of members of the cyclin dependent kinase inhibitor (CKI) CIP/KIP family, including p21^CIP1/WAF1^ and p27^KIP1^ [14, 16, 19, 61, 62], the licensing factors Cdt1 [63–65], and Set8 [22, 66–68]. Here, we observed that expression of *p21a/b* ^*CIP1/ WAF1*^ was dramatically increased in zebrafish *ddb1* ^*m863*^ mutants, whereas *p27a/b* ^*kIP1*^ and *p57* ^*kIP2*^ expression was not altered. These results suggest that loss of Ddb1 may also lead to enhanced *p21b* ^*CIP1/WAF1*^ transcript levels. Previous studies have already shown that p21^CIP1/WAF1^ is dramatically accumulated in the epidermis of DDB1-deficient mice [28]. Since DDB1 can transcriptionally regulate UV-induced genes [69, 70] and may function as a transcriptional partner of E2F1 [71], it could be speculated that Ddb1 is not only involved in the post-translational regulation of *p21* ^*CIP1/ WAF1*^ family members, but also in their transcriptional regulation as observed in our zebrafish *ddb1* ^*m863*^ mutants. However, since *p21* ^*CIP1/WAF1*^ is a well-established direct transcriptional target of p53 [51, 72], and *p53* transcription was upregulated in *ddb1* ^*m863*^ mutants, it seems more likely that the transcriptional activation of *p21a/b* ^*CIP1/ WAF1*^ may be mediated through elevated p53 levels in *ddb1* ^*m863*^ mutants. In addition, the p53 repressed genes *CCNA2* [73] and *CCND1* [74] were also downregulated in *ddb1* ^*m863*^ mutants. Further, the DDB1-CUL4A complex together with PCNA interact physically with p53 and the inactivation of this complex leads to p53 stabilization and cell cycle arrest *in vitro* [75]. This supports the idea that the altered transcriptional regulation of cell cycle genes in *ddb1* ^*m863*^ mutant embryos may be mediated by the upregulation of *p53*.

In summary, our studies revealed the requirement of Ddb1 for proper cell cycle progression and survival of proliferating cells in our zebrafish *in vivo* model. Previous studies showed that DDB1 guards the genomic stability in the cell division cycle by its DNA repair function, and coordinates cell cycle progression through the DDB1-CUL4A complex [76, 77], while loss of DDB1 leads to apoptosis and loss of genome integrity by regulating the activity of downstream targets in mice [25, 28]. Thus our data, together with the findings in mice, corroborate the concept of a ubiquitous requirement for DDB1 to maintain the correct cell division cycle and survival of proliferating cells. In this process, DDB1 may directly and indirectly regulate the proper activity of targets, at both post-transcriptional and transcriptional levels. Our zebrafish data also strengthen the notion of an involvement of Ddb1 in the transcriptional regulation of *p53* (Fig 9). Loss of DDB1 in other systems has been shown to lead to p53 protein stabilization [28]. Transcriptional upregulation of *p53* may be caused either by inactivation of the DDB1-CUL4A complex or by a stress signal from accumulated DNA damage (genomic instability). DNA damage can activate checkpoint kinases including Ataxia-Telangiectasia mutated (ATM) and Ataxia-Telangi-ectasia (ATR) [78–80], which have highly conserved and interconnected functions [81–83], and may affect p53 [42], we cannot excluded that upregulation of *p53* in *ddb1* ^*m863*^ mutants might be caused by interference with these signaling networks. In turn, the p53 activation/upregulation may lead to cell cycle arrest by upregulating CKIs and down regulating cyclins. Furthermore, the accumulation of p53 initiates the apoptosis pathway (Fig 9). The zebrafish *ddb1* ^*m863*^ mutant offers a new *in vivo* model to study the function of DDB1 and could contribute to unravel mechanisms by which DDB1 malfunction may contribute to neurodegeneration, aging, or carcinogenesis.

## Materials and Methods

### Fish strains

The zebrafish strains used in this project were the wild type (WT) strains AB, EK, WIK, and IND for mutagenesis and genetic mapping, ABTL and AB for expression analysis and knockdown experiments, and the mutant line carrying the *m863* allele. Embryos were obtained by natural spawning and raised at 28.5°C with a 14-hour light/10-hour dark cycle. Developmental stages were determined both by developmental time (hours/days post fertilization, hpf/dpf) and morphological characterization [84]. Melanin pigment formation was inhibited by incubating embryos in egg-water containing 0.2 mM 1-phenyl-2-thiourea (PTU, Sigma) [85]. The use of zebrafish in this research was approved by Regierungspräsidium Freiburg AZ 35–9185.81/G-12/49.

### Genomic DNA preparation from zebrafish

Genomic DNA was prepared from individual whole zebrafish embryos or from fin-clips. Briefly, zebrafish embryos or fins biopsies were transferred into 96-well PCR plate containing lysis buffer (10 mM Tris-Cl, pH = 8.0; 50 mM KCl; 0.3% Tween 20; 0.3% NP40; 1 mM EDTA) plus proteinase K (3.4 mg/ml). The plate was sealed with film (Microseal “A” film, Bio-Rad, USA) and samples were digested at 50°C overnight in a humid chamber. Then, Proteinase K was inactivated by heating samples for 10 min at 95°C. The resulting genomic DNA was stored at -80°C and diluted in ddH_2_O or 0.1xTE at proper ratio for PCR reaction.

### Mapping, cloning and genotyping of *ddb1* ^*m863*^

A genetic map cross was carried out between AB/EK strain carriers of the *m863* allele with WIK or IND wild type lines (G0) as previous described [86]. Initial mapping was performed using the bulked segregant analysis [87]. Potentially linked markers were confirmed by PCR on single map-cross embryos, and significance of a potential linkage was assumed values of logarithm of odds (LOD) scores higher than 3. High resolution mapping was performed by testing additional SSLP markers to define the critical region. Additional new polymorphic markers within the critical region were generated using the zebrafish SSR online tools (http://danio.mgh.harvard.edu/markers/ssr.html). The candidate gene *zgc*: *63840* (*ddb1*) was cloned from *in vitro* transcribed cDNA of individual two-day-old zebrafish by PCR (primer pairs see S1 Table). The *ddb1* ^*m863*^ embryos were genotyped by genomic PCR using a common reverse primer and mismatch forward primers that hybridize preferentially with mutant or wild type sequences (wild type forward primer, 5'-TGGTGGACCAGCACACGTTTGAGGG-3'; mutant forward primer, 5'-TGGTGGACCAGCACACGTTTGAGCC-3'; reverse primer, 5'-CACCGTCAGTATAGTGGAAGACGATGATGC-3').

### In situ hybridization and apoptosis assay

Whole-mount in situ hybridization (WISH) was performed as described [88]. The gene specific probe fragments, including tumor protein *p53* (NM_131327.1), *cyclin-dependent kinase inhibitor 1A* (*p21a/b* ^*CIP1/WAF1*^, OTTDART00000030315), *cyclin A2* (*ccna2*, NM_152949.1), *cyclin D1* (*ccnd1*, NM_131025.3), and *ddb1* (*zgc*: *63840*), were obtained by PCR (primer pairs see S1 Table) from cDNA of 2 dpf zebrafish wild type embryos, cloned into pCRII-TOPO plasmid, and verified by sequencing. The resulting plasmids pCRII-*p21a/b*, pCRII-*p53*, pCRII-*ccna2*, pCRII-*ccnd1*, and pCRII-*ddb1*, as well as pBS-II-*ccne2*, pBS-II-*p57* ^KIP2^ (Zebrafish International Resource Center (ZIRC), Oregon), and plasmids containing *pcna* [40] and *p27a/b* ^*KIP1*^ [89] were used for synthesis of Digoxiginin-labelled riboprobes (DIG RNA-labelling reagents, Roche Biochemicals). Apoptosis of zebrafish embryos was detected by 3´-end labeling of DNA fragments *in situ* using the ApopTag *In Situ* Apoptosis Detection Kit (S7100, Chemicon) as previously described [90] Whole mount stained embryos were sectioned using a vibratome as described [90].

### Microinjection

To generate synthetic capped Tol2 transposase mRNA, we used the pCS2FA-transposase plasmid [91] as template (mMessage mMachine kit, Ambion). To make *ddb1_egfp* fusion construct, a *ddb1* fragment of 220bp 5´ UTR nucleotides (primer see S1 Table) containing the *ddb1*_MO2 ATG Morpholino targeting sequence was cloned in frame upstream of the EGFP ORF. The construct was confirmed by sequencing

The Morpholinos *ddb1*_MO1 (5´---CCACCCTAAAGTGTGCTCACCTGGA---3), *ddb1*_MO2 (5---CGGTCACCACGTAGTTGTAGGACAT---3), and *p53*_MO (`)[92] were obtained from GeneTools (Philomath, USA). The mRNAs and DNA injection solutions were prepared in H_2_O containing 0.05% phenol red. The injection solutions were loaded into glass capillaries using Microloader Pipettes (Eppendorf) just prior to the injection. The injection volume was controlled by measuring the diameter of one drop of injection solution in halocarbon oil (Halocarbon Products Corporation, Series 27) on a micrometer slide. Embryos at one-cell stage were used for injection.

## Supporting Information

S1 Fig Multiple sequence alignment of DNA damage-binding protein 1 from six vertebrates. The DDB1 protein sequences used for alignment are from human (NP_001914.3), Chimpanzee (XP_508472.2), Bovine (NP_001073731.1), mouse (NP_056550.1), rat (NP_741992.1), chicken (NP_989547.1), and zebrafish (AFI92852.1, this work). Three β-propeller domains of BPA, BPB and BPC and a C-terminal domain CTD were marked according to the crystal structure of human DDB1 protein [10]. *Homo*, *Homo sapiens*; *Pan*, *Pan troglodytes*; *Bos*, *Bos taurus*; *Mus*, *Mus musculus*; *Rattus*, *Rattus norvegicus*; *Gallus*, *Gallus gallus*; *Danio*, *Danio rerio*.
(TIF)

S2 Fig Evaluation of the knockdown efficiency of *ddb1* morpholinos. (A-B) RT-PCR to test the efficiency of *ddb1* splice site targeted morpholino (*ddb1*_MO1). (A) Schematic diagram of *ddb1*_MO1 targeting the 5^th^ exon-intron junction. (B) Efficiency assay of *ddb1*_MO1 by RT-PCR using two pairs of *ddb1*-specific primers. The amount of RNA and cDNA used for RT-PCR was the same for the different samples and for the internal control *β-actin*. Negative control (con) contained no cDNA. (C-D) Evaluation of the knockdown efficiency of *ddb1* translation start site morpholino (*ddb1*_MO2). (C) Schematic representation of *ddb1*-*egfp* fusion construct used for *ddb1*_MO2 efficiency assay. (D) The expression of EGFP in larvae injected with *ddb1*-*egfp* fusion construct together with or without *ddb1*_MO2. Anterior towards the left. Abbreviations used: E4/E5/E6, 4^th^ /5^th^ /6^th^ exon; I4/I5/I6, 4^th^ / 5^th^ / 6^th^ intron; *ddb1*_LF/R, the forward and reverse primers of longer *ddb1*-specific fragment; *ddb1*_SF/R, forward and reverse primers of shorter *ddb1*-specific fragment; 1 and 2, different samples; uninj, uninjected embryos.
(TIF)

S3 Fig Dopaminergic phenotype in zebrafish larvae injected with *ddb1*_MO1. (A-I) The *th* expression pattern in wild type control larvae (A-C), in larvae injected with *ddb1*_MO1 (4 ng/embryo) (D-F), and larvae injected with *ddb1*_MO1 (4 ng/embryo) and the same amount of *p53*_MO (G-I) was analyzed by WISH. (A, D, G) lateral views and (B-C, E-F, H-I) dorsal views. Red arrows point at affected *th*-expressing neurons in the pretectum (D-E, G-H) and retina (F, I). Abbreviations used: AAN, arch-associated neurons; H, hypothalamus; LC, locus coeruleus; MO, medulla oblongata; OB, olfactory bulb; Pr, pretectum; PT, posterior tuberculum; SP, subpallium; sym, sympathetic neurons; VT, ventral thalamus. Anterior towards the left. Scale bar: 100 μm.
(TIF)

S4 Fig Characterization of *ddb1* expression pattern during wild type zebrafish embryonic development by WISH and RT-PCR. (A-I') The WISH signal of *ddb1* antisense probe (A-I) and its sense control (A'-I') in embryos before 24 hpf. *ddb1* transcript was ubiquitously detected in all blastomeres (A-F') before mid-blastula transition (MBT) when zygotic transcription starts, revealing that *ddb1* was expressed maternally. The ubiquitous expression was continued in subsequent stages including sphere, shield and 80% epiboly (G-I, G'-I'). (J-R) The expression of *ddb1* at 24 hpf (J-L), 48 hpf (M-O), and 72 hpf (P-R). From 24 hpf onwards, *ddb1* mRNA was observed to be expressed broadly and at high levels in the brain and somites (J-L), then spatially restricted to the brain, retina (high in the CMZ but moderate in the GCL and INL, M), the branchial arches, and endoderm at 48 hpf (N-O), followed at 72 hpf by downregulation and more distinct spatial expression pattern (P-R). *ddb1* was detected at moderate levels in the telencephalic proliferation region, tectal proliferation region, cerebellum, CMZ, and branchial arches, whereas in other regions the signal was weak (P-Q). (S) *ddb1* was maternally expressed as revealed by RT-PCR from two separate cDNA templates prepared from one cell stage zygotes (lanes 1 and 2; lane 3—control with water only as template) using a *ddb1* specific primer pair.
(TIF)

S5 Fig Reduction of *ddb1* mRNA in homozygous *ddb1* ^*m863*^ mutants by WISH and RT-PCR. (A-I) WISH analysis of *ddb1* transcripts revealed reduced expression in heterozygous (B, E, H) and near-absent expression in homozygous *ddb1* ^*m863*^ mutants (C, F, I) compared to wild type siblings (A, D, G) at 24 hpf (A-C) and 72 hpf (D-I). Planes focus on the retina of heterozygous embryos (B') and homozygous mutants (C'). Lateral views (A-C; G-I) and dorsal views (D-F). Abbreviations used: Cce, cerebellum; dc, diencephalon; hb, hindbrain; j, jaw; I, lens; li, liver; mb, midbrain; ret, retina; sc, spinal cord; T, tectum; tc, telencephalon. Anterior is towards the left. Scale bars in C' for B'-C', in I for others: 100 μm. (J) RT-PCR analysis of *ddb1* transcript levels during development stages from 24 hpf to 96 hpf in wild type, heterozygous and homozygous *m863* mutants. *β-actin* was used as an internal control, and the negative control was template-free. (K) The RT-PCR gel was quantified densitometrically and traces of *ddb1* PCR signal and of internal *β-actin* control were shown together with the wild type, heterozygous and homozygous *m863* mutant samples adjacent to each other within each frame showing one developmental stage. Transcription of *ddb1* was progressively downregulated in wild type embryos along the development. Compared to wild type siblings, the transcription level of *ddb1* was slightly decreased in heterozygous embryos/larvae, but strongly reduced in homozygous *ddb1* ^*m863*^ mutants.
(TIF)

S1 Table Primer pairs for cloning of ddb1 and other riboprobe fragments. (PDF)

S2 Table DDB1 from different vertebrate species. (PDF)
