# Supplementary figures and images for: Loss of DDB1 Leads to Transcriptional p53 Pathway Activation in Proliferating Cells, Cell Cycle Deregulation, and Apoptosis in Zebrafish Embryos
Source: PLoS One. 2015 Jul 30;10(7):e0134299. doi: 10.1371/journal.pone.0134299 (PMC4520591; doi:10.1371/journal.pone.0134299)

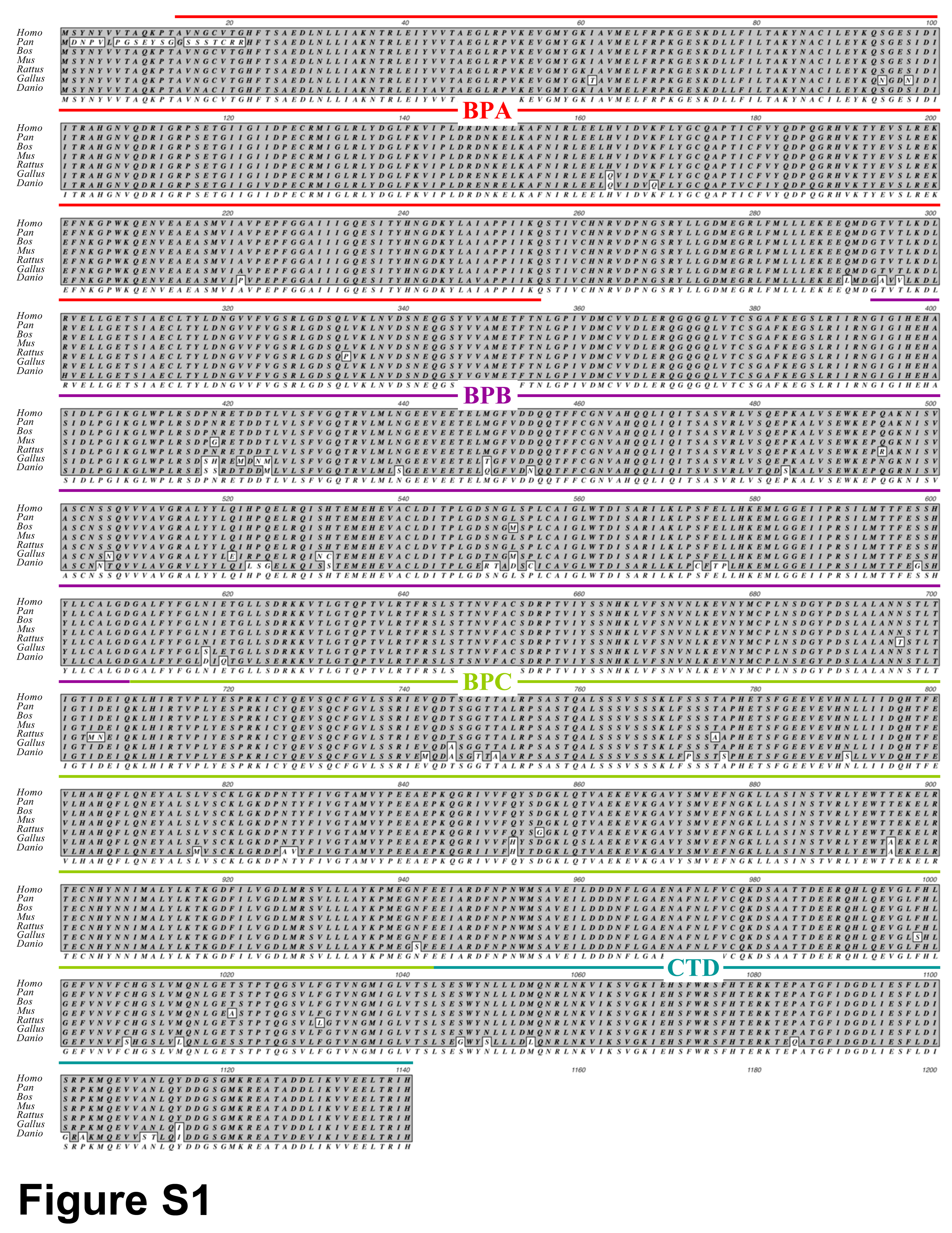

Supplement: S1 Fig — The DDB1 protein sequences used for alignment are from human (NP_001914.3), Chimpanzee (XP_508472.2), Bovine (NP_001073731.1), mouse (NP_056550.1), rat (NP_741992.1), chicken (NP_989547.1), and zebrafish (AFI92852.1, this work). Three β-propeller domains of BPA, BPB and BPC and a C-terminal domain CTD were marked according to the crystal structure of human DDB1 protein [10]. Homo, Homo sapiens; Pan, Pan troglodytes; Bos, Bos taurus; Mus, Mus musculus; Rattus, Rattus norvegicus; Gallus, Gallus gallus; Danio, Danio rerio. (TIF) [file pone.0134299.s001.tif]

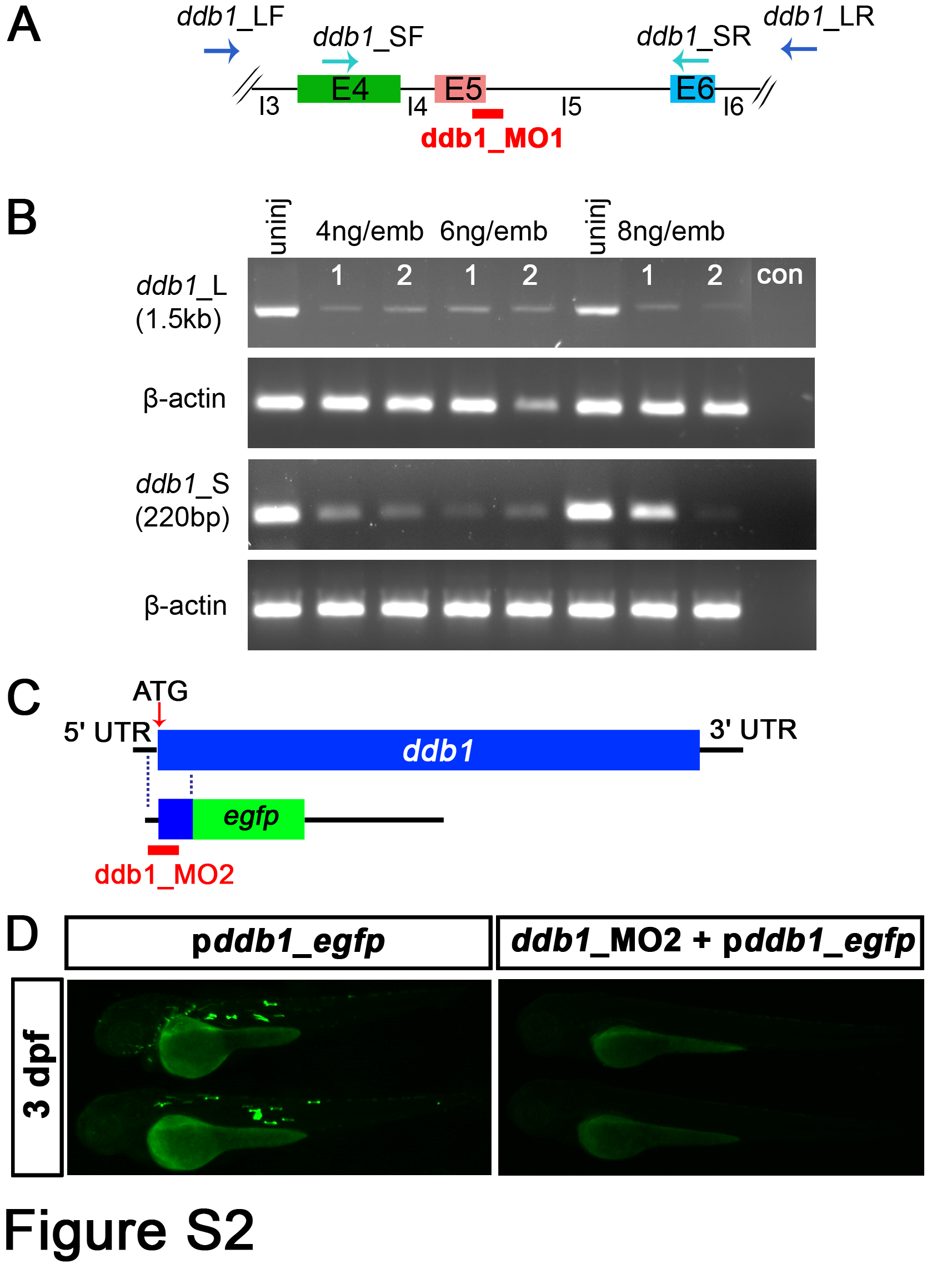

Supplement: S2 Fig — (A-B) RT-PCR to test the efficiency of ddb1 splice site targeted morpholino (ddb1_MO1). (A) Schematic diagram of ddb1_MO1 targeting the 5th exon-intron junction. (B) Efficiency assay of ddb1_MO1 by RT-PCR using two pairs of ddb1-specific primers. The amount of RNA and cDNA used for RT-PCR was the same for the different samples and for the internal control β-actin. Negative control (con) contained no cDNA. (C-D) Evaluation of the knockdown efficiency of ddb1 translation start site morpholino (ddb1_MO2). (C) Schematic representation of ddb1-egfp fusion construct used for ddb1_MO2 efficiency assay. (D) The expression of EGFP in larvae injected with ddb1-egfp fusion construct together with or without ddb1_MO2. Anterior towards the left. Abbreviations used: E4/E5/E6, 4th /5th /6th exon; I4/I5/I6, 4th / 5th / 6th intron; ddb1_LF/R, the forward and reverse primers of longer ddb1-specific fragment; ddb1_SF/R, forward and reverse primers of shorter ddb1-specific fragment; 1 and 2, different samples; uninj, uninjected embryos. (TIF) [file pone.0134299.s002.tif]

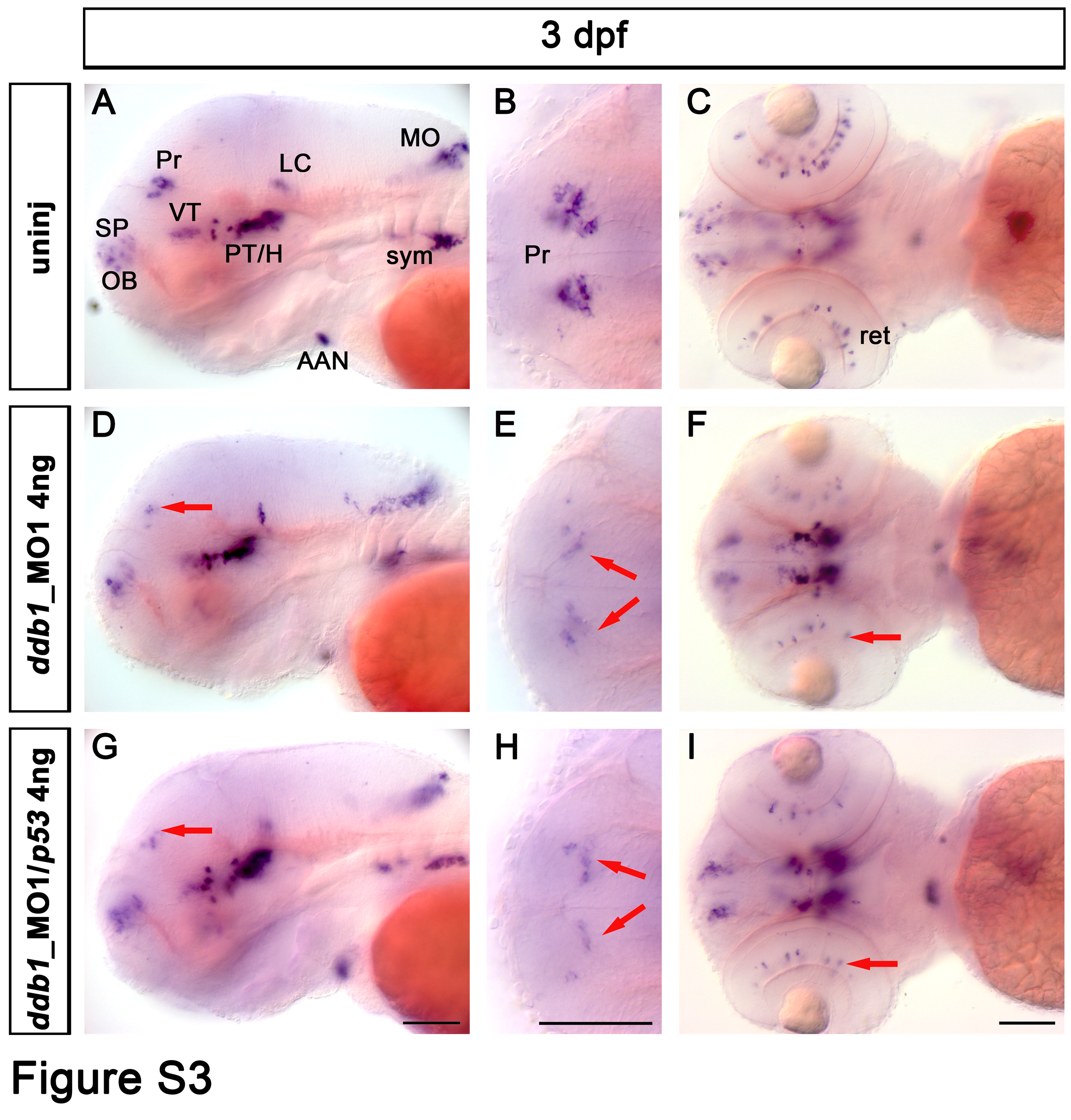

Supplement: S3 Fig — (A-I) The th expression pattern in wild type control larvae (A-C), in larvae injected with ddb1_MO1 (4 ng/embryo) (D-F), and larvae injected with ddb1_MO1 (4 ng/embryo) and the same amount of p53_MO (G-I) was analyzed by WISH. (A, D, G) lateral views and (B-C, E-F, H-I) dorsal views. Red arrows point at affected th-expressing neurons in the pretectum (D-E, G-H) and retina (F, I). Abbreviations used: AAN, arch-associated neurons; H, hypothalamus; LC, locus coeruleus; MO, medulla oblongata; OB, olfactory bulb; Pr, pretectum; PT, posterior tuberculum; SP, subpallium; sym, sympathetic neurons; VT, ventral thalamus. Anterior towards the left. Scale bar: 100 μm. (TIF) [file pone.0134299.s003.tif]

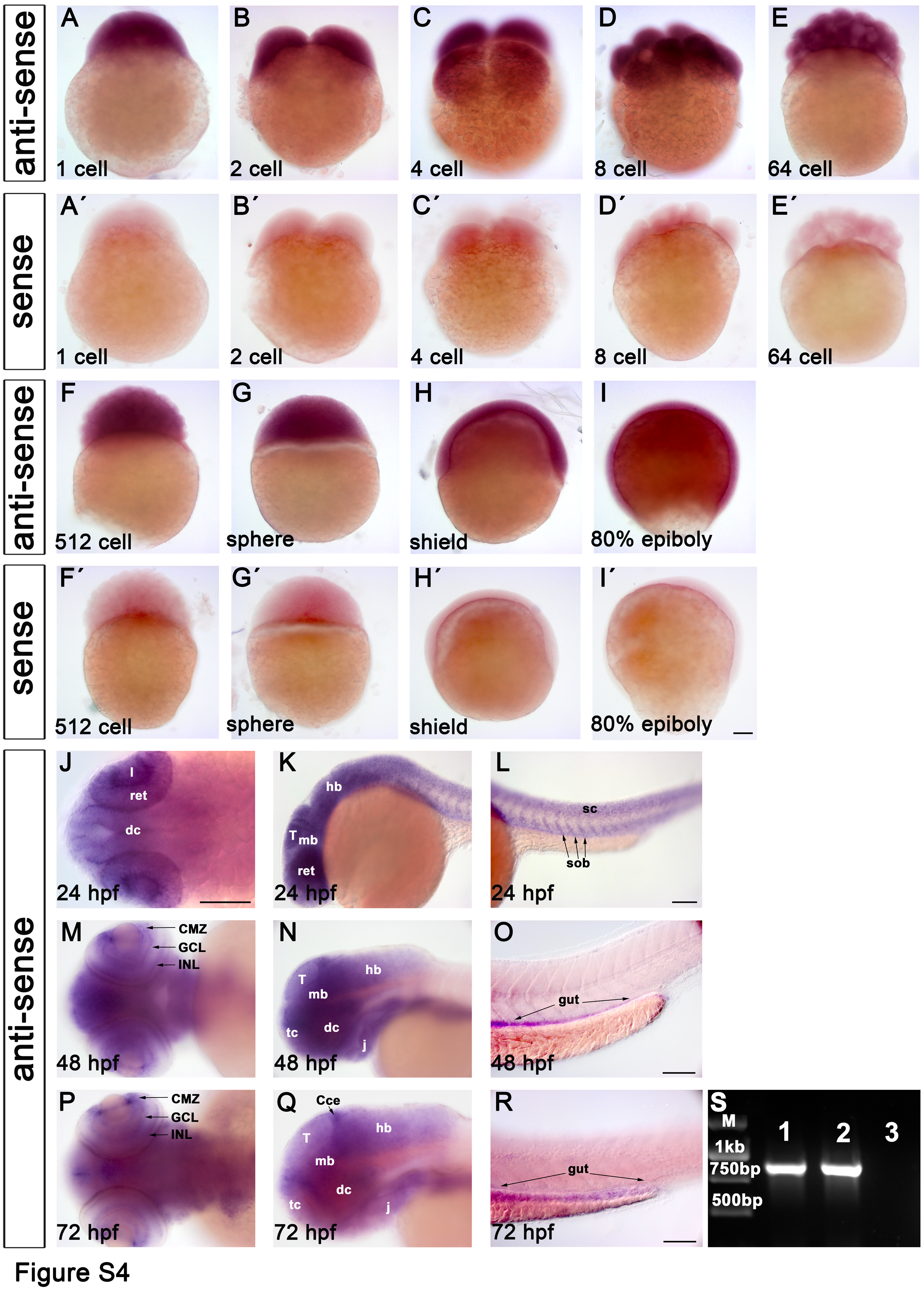

Supplement: S4 Fig — (A-I') The WISH signal of ddb1 antisense probe (A-I) and its sense control (A'-I') in embryos before 24 hpf. ddb1 transcript was ubiquitously detected in all blastomeres (A-F') before mid-blastula transition (MBT) when zygotic transcription starts, revealing that ddb1 was expressed maternally. The ubiquitous expression was continued in subsequent stages including sphere, shield and 80% epiboly (G-I, G'-I'). (J-R) The expression of ddb1 at 24 hpf (J-L), 48 hpf (M-O), and 72 hpf (P-R). From 24 hpf onwards, ddb1 mRNA was observed to be expressed broadly and at high levels in the brain and somites (J-L), then spatially restricted to the brain, retina (high in the CMZ but moderate in the GCL and INL, M), the branchial arches, and endoderm at 48 hpf (N-O), followed at 72 hpf by downregulation and more distinct spatial expression pattern (P-R). ddb1 was detected at moderate levels in the telencephalic proliferation region, tectal proliferation region, cerebellum, CMZ, and branchial arches, whereas in other regions the signal was weak (P-Q). (S) ddb1 was maternally expressed as revealed by RT-PCR from two separate cDNA templates prepared from one cell stage zygotes (lanes 1 and 2; lane 3—control with water only as template) using a ddb1 specific primer pair. (TIF) [file pone.0134299.s004.tif]

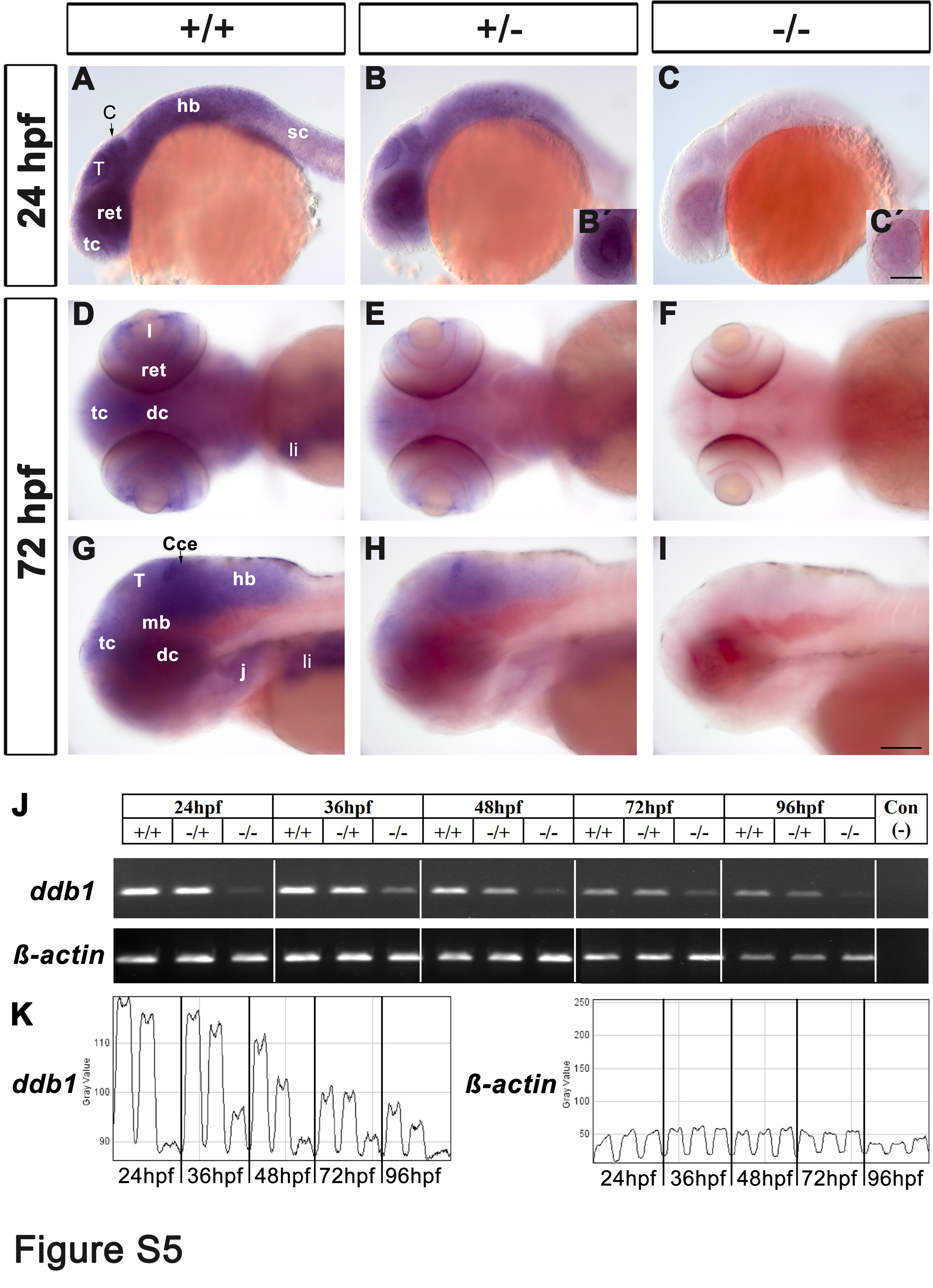

Supplement: S5 Fig — (A-I) WISH analysis of ddb1 transcripts revealed reduced expression in heterozygous (B, E, H) and near-absent expression in homozygous ddb1 m863 mutants (C, F, I) compared to wild type siblings (A, D, G) at 24 hpf (A-C) and 72 hpf (D-I). Planes focus on the retina of heterozygous embryos (B') and homozygous mutants (C'). Lateral views (A-C; G-I) and dorsal views (D-F). Abbreviations used: Cce, cerebellum; dc, diencephalon; hb, hindbrain; j, jaw; I, lens; li, liver; mb, midbrain; ret, retina; sc, spinal cord; T, tectum; tc, telencephalon. Anterior is towards the left. Scale bars in C' for B'-C', in I for others: 100 μm. (J) RT-PCR analysis of ddb1 transcript levels during development stages from 24 hpf to 96 hpf in wild type, heterozygous and homozygous m863 mutants. β-actin was used as an internal control, and the negative control was template-free. (K) The RT-PCR gel was quantified densitometrically and traces of ddb1 PCR signal and of internal β-actin control were shown together with the wild type, heterozygous and homozygous m863 mutant samples adjacent to each other within each frame showing one developmental stage. Transcription of ddb1 was progressively downregulated in wild type embryos along the development. Compared to wild type siblings, the transcription level of ddb1 was slightly decreased in heterozygous embryos/larvae, but strongly reduced in homozygous ddb1 m863 mutants. (TIF) [file pone.0134299.s005.tif]
