# Supplementary material for: Loss of DDB1 Leads to Transcriptional p53 Pathway Activation in Proliferating Cells, Cell Cycle Deregulation, and Apoptosis in Zebrafish Embryos
Source: PLoS One. 2015 Jul 30;10(7):e0134299. doi: 10.1371/journal.pone.0134299 (PMC4520591; doi:10.1371/journal.pone.0134299)
Supplement: S1 Table — (PDF) [file pone.0134299.s006.pdf]

S1 Table. Primer pairs for cloning of *ddb1* and other riboprobe fragments.

Name	Forward 5'------3'	Reverse 5'------3'
Primers for ddb1 cloning		
ddb1_c1	GAACGCCATGTCCTACAACTACG	CAGCAGGTACCGCGAGCC
ddb1_c2	CGCCCATTATCAAGCAAAGC	AGGTAGTACAGCACTCTGCCCA
ddb1_c3	AGGAACATCAGCGTGGCGT	CACCTCCTCCCCGAATGACG
ddb1_c4	CTCTGTCCAGCAGTGTGAGCTC	CTAACAGAAACATCACAATGAGGACG
*ddb1*_XhoF	AATACTCGAGgaacgccatgtcctacaactacg	
*ddb1*_KpnR		AATAGGTACCTCTCCCTTGGGCCTGAACAG
Primers for riboprobe fragments		
ccna2	GGAAGGATGTCAACACAAGGAAG	GAGAGAACTGTCAGCACCAGATG
ccnd1	TTTGCTGCGAAGTGGATACCA	AACAGTTTGGGCGTGCTGAGT
p21b	TCCTACGTTCACTCGGTAATGG	TGAGAACTTACTGGCAGCTTCA
p53	GGTGGTGGACGTTGCCCCTC	GCAGTTCCCCAAATGACCCCTGT
ddb1	AACAGCACTCTCACCATCGGC	ATAGGCCAGCAGCAGCACG
