# Supplementary material for: Loss of DDB1 Leads to Transcriptional p53 Pathway Activation in Proliferating Cells, Cell Cycle Deregulation, and Apoptosis in Zebrafish Embryos
Source: PLoS One. 2015 Jul 30;10(7):e0134299. doi: 10.1371/journal.pone.0134299 (PMC4520591; doi:10.1371/journal.pone.0134299)
Supplement: S2 Table — (PDF) [file pone.0134299.s007.pdf]

S2 Table. DDB1 from different vertebrate species

Species	Gene symbol	Protein symbol	mRNA ID	Protein ID	Length
H.sapiens	*DDB1*	DDB1	NM_001923.3	NP_001914.3	1140aa
R.norvegicus	*Ddb1*	DDB1	NM_171995.1	NP_741992.1	1140aa
P.troglodytes	*DDB1*	DDB1	XM_508472.2	XP_508472.2	1140aa
B.taurus	*DDB1*	DDB1	NM_001080262.1	NP_001073731.1	1140aa
M.musculus	*Ddb1*	DDB1	NM_015735.1	NP_056550.1	1140aa
G.gallus	*DDB1*	DDB1	NM_204216.2	NP_989547.1	1140aa
D.rerio	*zgc:63840*		NM_200626.1	NP_956920.1	897aa
D.rerio	*ddb1*	Ddb1	JQ692623.1	AFI92852.1	1140aa
